# Fusion assays for screening of fusion inhibitors targeting SARS-CoV-2 entry and syncytia formation

**DOI:** 10.3389/fphar.2022.1007527

**Published:** 2022-11-11

**Authors:** Shiu-Wan Chan

**Affiliations:** Faculty of Biology, Medicine and Health, School of Biological Sciences, The University of Manchester, Manchester, United Kingdom

**Keywords:** SARS-CoV-2, COVID-19, fusion, fusion assay, drug screening, fusion inhibitor, syncytia, virus entry

## Abstract

Virus fusion process is evolutionarily conserved and provides a promising pan-viral target. Cell-cell fusion leads to syncytial formation and has implications in pathogenesis, virus spread and immune evasion. Drugs that target these processes can be developed into anti-virals. Here, we have developed sensitive, rapid, adaptable fusion reporter gene assays as models for plasma membrane and alternative fusion pathways as well as syncytial fusion in the severe acute respiratory syndrome coronavirus 2 (SARS-CoV-2) and have confirmed their specificity using neutralizing antibodies and specific protease inhibitors. The fusion report gene assays are more sensitive and unbiased than morphological fusion assay. The fusion assays can differentiate between transmembrane serine protease 2 (TMPRSS2)-dependency in TMPRSS2(+) cells and trypsin-dependency in angiotensin-converting enzyme 2 (ACE2)(+)TMPRSS2(-) cells. Moreover, we have identified putative novel fusion processes that are triggered by an acidic pH with and without trypsin. Coupled with morphological fusion criteria, we have found that syncytia formation is enhanced by TMPRSS2 or trypsin. By testing against our top drug hits previously shown to inhibit SARS-CoV-2 pseudovirus infection, we have identified several fusion inhibitors including structurally related lopsided kite-shaped molecules. Our results have important implications in the development of universal blockers and synergistic therapeutics and the small molecule inhibitors can provide important tools in elucidating the fusion process.

## Introduction

The coronavirus disease 2019 (COVID-19) pandemic has caused unprecedented disruption and severely paralysed the society and economy and put the global health under tremendous stress (Huang et al., 2020). The severe acute respiratory syndrome coronavirus 2 (SARS-CoV-2) is the aetiological agent of the COVID-19. At the beginning of the pandemic, the rapid development of several vaccines has reduced disease severity and hospitalization but they do not induce sufficient immunity to prevent infection (Juthani et al., 2021; Tenforde et al., 2021). This is exacerbated by the emergence of SARS-CoV-2 variants (alpha, beta, gamma, delta, omicron) later in the pandemic, which is an inevitable outcome of virus evolution (Telenti et al., 2022). Of particular concern is the ability of the later variants to evade protective immunity from vaccination and previous infections (Arora et al., 2022). The continual evolution of the current pandemic virus is posing problems to preventative measures and treatments against single targets (Callaway, 2022). Emergence of pandemic viruses and viruses with pandemic potential (swine flu, bird flu, SARS-CoV, Zika virus) has been accelerated during the last 20 years owing to climate change, deforestation, urbanization and international travel (Chan, 2002; Hawkey et al., 2003; Basarab et al., 2016; Huang et al., 2020; Ou et al., 2021). Hence, there is an urgent need to find a broad-spectrum anti-viral as a first line defence against the current and future pandemics.

Fusion is one such potential universal viral target (Eckert and Kim, 2001). The spike protein is divided into the S1 and S2 subunits (Hoffmann et al., 2020; Walls et al., 2020). The S1 subunit contains the receptor binding domain (RBD) and receptor binding motif (RBM) and is less conserved than the S2 subunit which contains the highly conserved fusion domain and fusion peptide (Chan, 2020). Virus fusion is mediated by one of two pathways: plasma membrane fusion and endosomal membrane fusion (Eckert and Kim, 2001). Normally, viruses have evolved to use one of the pathways. SARS-CoV-2 and some coronavirus family members are unusual in that they can employ one or the other fusion pathway (Belouzard et al., 2012; Ou et al., 2021). The pre-requisite of coronavirus fusion is cleavage between the S1 and S2 (S1/S2 site) and the downstream S2′ site (Hoffmann et al., 2020; Ou et al., 2020; Walls et al., 2020). The multi-basic S1/S2 site of the SARS-CoV-2 spike protein is cleaved by the ubiquitous furin during trafficking through the Golgi apparatus, hence, virions egress from the host cells are already equipped with cleaved S1 and S2 subunits (Walls et al., 2020). Therefore, only one cleavage at the S2′ is required to expose the fusion peptide. As a result, the availability of S2′ protease in the micro-environment dictates the location of fusion and virus entry. Binding of the S1 subunit to the angiotensin-converting enzyme 2 (ACE2) receptor is essential in priming fusion (Yu et al., 2022). The transmembrane serine protease 2 (TMPRSS2) mediates S2′ cleavage to enable plasma membrane fusion (Hoffmann et al., 2020). Otherwise, the virion is endocytosed and trafficked to the endosome in which the endosomal cathepsin L mediates S2’ cleavage to enable endosomal fusion (Ou et al., 2020). In analogy to SARS-CoV, SARS-CoV-2 depends on an acidic pH in the endosome to activate the cathepsin L, rather than to cause conformational changes in the S2 domain, as is common in other acidic pH-dependent viruses (Simmons et al., 2005; Ou et al., 2020). Hence, an acidic pH is not strictly required if the protease has been pre-activated.

The S2 fusion domain contains a fusion peptide and the heptad repeat 1 (HR1) and HR2 (Chan, 2020). S2’ cleavage will release the now terminal, hydrophobic fusion peptide to insert into the host plasma membrane. Interactions between three HR1 and three HR2 results in folding back of the fusion domain to form a six-helix bundle. This brings the viral and host membrane into proximity to allow hemifusion and pore formation to occur to release the nucleocapsid into the cytoplasm.

In addition to being essential in virus entry, leaky transport of the spike protein to the plasma membrane can cause fusion of neighbouring cells to form giant cells (syncytia). Syncytia formation is an important phenomenon of pathogenesis, inter-cell virus spread, and immune escape in a number of virus infections e.g. the human immunodeficiency virus (HIV), the respiratory syncytial virus (Leroy et al., 2020). Syncytia formation has been observed in COVID-19 patient lung tissues (Bussani et al., 2020; Tian et al., 2020; Xu et al., 2020; Braga et al., 2021).

Targeting fusion traditionally focuses on using synthetic HR2 analogue to disrupt HR1 interaction, after the first clinically approved fusion inhibitor, enfuvirtide, in HIV treatment (Poveda et al., 2005). In this case, inhibition is specific for single viruses. Understanding the fusion process will generate new druggable targets. Small molecules present a new avenue to target the fusion process beyond the HR1 (Chan, 2020). In this study, we aimed to set up surrogate fusion assays in drug screening and to facilitate investigation into virus-cell fusion process and syncytia formation.

## Materials and methods

### Cell culture

293T, 293T-ACE2, 293T-ACE2-TMPRSS2, Vero and Huh-7 cells were cultured in Dulbecco’s modified Eagle’s medium with 4 mM glutamate (DMEM; Sigma) and supplemented with 10% fetal calf serum (FCS; Sigma), 100 units/ml penicillin and 100 μg/ml streptomycin (Sigma) at 37°C, 5% CO_2_. Huh-7 was supplemented with 1× non-essential amino acids.

### Plasmids

**FIGURE 1:**
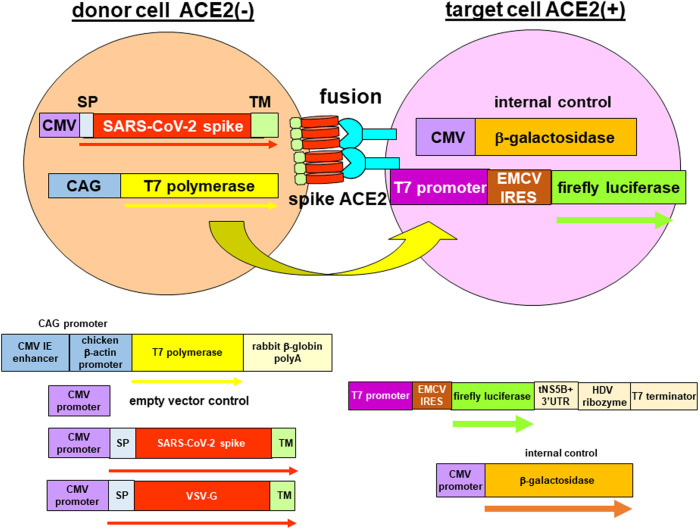
Reporter gene fusion assay. Donor cells (293T) were co-transfected with plasmids encoding the T7 polymerase together with either the empty vector (negative control) or a viral envelope protein i.e., severe acute respiratory syndrome coronavirus 2 (SARS-CoV-2) spike protein or vesicular stomatitis virus glycoprotein (VSV-G). The viral envelope proteins contain authentic signal peptides and transmembrane domains to target to the plasma membrane. Although the SARS-CoV-2 spike protein was localized to the endoplasmic reticulum-Golgi intermediate compartment, leaky transport would still target a proportion of the spike protein to the plasma membrane. Target cells (either 293T-ACE2 or 293T-ACE2-TMPRSS2) were co-transfected with two reporter genes expressing the luciferase protein under the T7 promoter and the internal control, β-galactosidase under the CMV promoter. Binding of the spike protein with the angiotensin-converting enzyme 2 (ACE2) protein will trigger fusion of the donor and target cells. Mixing of cytoplasmic contents enabled the T7 polymerase from the donor cells to bind to the T7 promoter in the target cells to drive transcription of the luciferase gene. The transcript was then translated from the encephalomyocarditis (EMCV) internal ribosome entry site (IRES) element to produce a luciferase read-out. Fusion was measured as a ratio of the luciferase activity to the β-galactosidase activity.

The plasmid T7EMCVLuc contains the luciferase reporter gene under the T7 promoter and the encephalomyocarditis virus (EMCV) internal ribosome entry site (IRES) element and terminated with a T7 terminator (Figure 1) (Aoki et al., 1998). Because the T7 polymerase does not have capping ability and localized in the cytoplasm, an EMCV IRES element is used to translate the luciferase gene. The luciferase gene is cleaved from the truncated hepatitis C virus (HCV) non-structural 5B (NS5B) and 3′ untranslated region (UTR) by the hepatitis D virus (HDV) ribozyme to produce authentic 3′ end. The plasmid CMVβgal contains the β-galactosidase reporter gene driven by the cytomegalovirus (CMV) promoter. The plasmid CAGT7 contains the T7 polymerase gene transcribed by the CMV immediate-early (IE) enhancer and chicken β-actin promoter (CAG promoter) and ends in rabbit β-globin polyA. The plasmid pcDNA3.1 is the empty plasmid vector. The plasmids CMVSARS1S and CMVSARS2S contain the SARS-CoV spike protein (SARS-1-S) and SARS-CoV-2 spike protein (SARS-2-S) genes, respectively, encompassed by their authentic signal peptide and transmembrane domain. The plasmid CAGVSVG contains the vesicular stomatitis virus glycoprotein (VSV-G) gene encompassed by its authentic signal peptide and transmembrane domain under the CAG promoter.

### Reporter gene fusion assay

293T/293T-ACE2/293T-ACE2-TMPRSS2 cells were transfected in either 100 mm dishes or 6-well plates at seeding densities of 4 × 10^6^ cells/100 mm dish or 7.2 × 10^5^ cells/well of a 6-well plate in 10% FCS/DMEM without antibiotics. Cells were transfected using calcium phosphate (125 mM CaCl_2_, 0.7 mM Na_2_HPO_4_, 70 mM NaCl, 25 mM (4-(2-hydroxyethyl)-1-piperazineethanesulfonic acid) (HEPES) pH 7.05, as described previously (Chan et al., 2021). Donor 293T cells were co-transfected with CAGT7 and either the pcDNA3.1, CMVSARS1S, CMVSARS2S or CAGVSVG in a 1:1 ratio (CAGVSVG was made up of 1/10th CAGVSVG and 9/10th pcDNA3.1). Target cells (293T-ACE2 or 293T-ACE2-TMPRSS2) were co-transfected with T7EMCVLuc and CMVβgal in a 3:1 ratio. Vero cells were seeded at 4 × 10^5^ cells/well of a 6-well plate in 10% FCS/DMEM without antibiotics. Vero cells were co-transfected with T7EMCVLuc and CMVβgal in a 3:1 ratio using Lipofetamine 3000 Reagent (Invitrogen) according to the manufacturer’s instructions. Briefly, 2 μg of DNA were diluted in 100 μl of Opti-MEM medium (Invitrogen) and mixed with 4 μl of P3000 Reagent and then added to 6 μl of Lipofetamine 3000 Reagent which had been pre-mixed with 100 μl of Opti-MEM medium. The following day, transfected cells were detached using non-enzymatic dissociation agent (Sartorius) and re-seeded at 19,000 cells per well of a 96-well plate for each of the donor and target cells in 10%FCS/DMEM supplemented with 25 mM HEPES to generate a physiological pH of 7.4. If drugs were tested for fusion inhibition, 10 μM of individual drugs diluted in DMEM/HEPES were added to pre-incubate with the target cells for an hour before addition of donor cells with drugs maintained at 10 μM. Leupeptin was used at 10 μg/ml. Soybean trypsin inhibitor (SBTi) was used at 75 μg/ml. Neutralizing antibodies were used at 10 μg/ml (ACROB SPD-M128) and 28 μg/ml (Sino Biol, 40592-R001), respectively. After an overnight of incubation, the co-culture was either untreated (physiological pH) or treated with trypsin and/or acidic pH. Treatment of pH7.4 + tryspin involved incubation of the co-culture in 15 μg/ml trypsin in DMEM/25 mM HEPES for 2min and neutralization with 75 μg/ml SBTi in an equal volume of 20%FCS/DMEM/25 mM HEPES. Treatment of pH5 involved incubation of the co-culture in pH5 fusion buffer (130 mM NaCl, 15 mM Na citrate, 10 mM morpholine ethanesulfonic acid, 5 mM HEPES; adjusted to pH5) for 2min followed by replacement of the fusion buffer with 10%FCS/DMEM/25 mM HEPES. Treatment of pH5+tryspin involved incubation of the co-culture in 15 μg/ml trypsin in pH5 fusion buffer for 2min followed by replacement of the fusion buffer with 75 μg/ml SBTi in 10%FCS/DMEM/25 mM HEPES. If drugs were tested for fusion inhibition, their concentrations would be maintained during the 2min treatment and post-treatment. After 5 h incubation at 37°C/5% CO_2_, cells were harvested for luciferase and β-galactosidase assays.

### Luciferase and β-galactosidase assays

Cells were lysed by the addition of 100 μl of passive lysis buffer (Promega/Biotium) to each well and shaken for >15min. Luciferase assay was carried out as described in (Chan and Egan, 2005; Chan and Egan, 2009; Egan et al., 2013; Chan et al., 2021) in a buffer containing 0.0165 M glycylglycine, 0.01 M MgSO_4_, 2.65 mM EGTA, 10.5 mM potassium phosphate, 1.4 mM adenosine 5′-triphosphate, 0.86 mM dithiothreitol (DTT), 0.175 mg/ml bovine serum albumin (BSA), and 0.035 mM luciferin (Promega) using 50 μl of the lysate and a luminometer (Berthold Technologies, Germany). A standard curve was constructed using recombinant luciferase (Promega). Beta-galactosidase assay was carried out as described in (Chan and Egan, 2005; Chan and Egan, 2009; Egan et al., 2013) in a buffer containing 100 mM sodium phosphate pH7.3, 1mM MgCl_2_, 50 mM β-mercaptoethanol and 0.665 mg/ml o-nitrophenyl β-D-galactopyranoside (Sigma) using 20 μl of the lysate and read at 420 nm using a plate reader (Bio-Tek Synergy HT). Luciferase activity was normalized against β-galactosidase activity.

### XTT viability assay

Cell viability of the fusion co-cultures (293T + 293T-ACE2 or 293T + 293T-ACE2-TMPRSS2) at physiological pH was measured as described in (Chan et al., 2021) by addition of 50 μl of 1 mg/ml 2,3-Bis- (2-Methoxy-4-nitro-5-sulfophenyl)-2H-tetrazolium-5-carboxanilide, disodium salt (XTT) (Biotium/Alfa Aezar) and 20 μM N-methyl dibenzopyrazine methyl sulfate (Cayman) in culture medium to each well for 2–4 h at 37°C/5% CO_2_. Absorbance was read at 450 nm with a reference wavelength of 650 nm using a plate reader (Bio-Tek Synergy HT).

### Negative screen

Cells (293T, 293T-ACE2 or 293T-ACE2-TMPRSS2) were co-transfected with CAGT7, T7EMCVLuc and CMVβgal in a 2:2:1 ratio using calcium phosphate (125 mM CaCl_2_, 0.7 mM Na_2_HPO_4_, 70 mM sodium chloride, 25 mM HEPES pH 7.05) for 24 h, re-seeded into 96-well plates at 30,000 cells/well for 6 h before 10 μM of individual drugs were added to incubate for a further 16–24 h and harvested for luciferase and β-galactosidase assays.

### Morphological fusion assay

Donor 293T cells were transfected with either the pcDNA3.1 or CMVSARS2S using calcium phosphate for 30 h. Target cells (293T-ACE2 or 293T-ACE2-TMPRSS2) were dissociated and re-seeded at 50,000 cells per well of a 96-well plate and pre-incubated with 10 μM of drugs for 1 h before addition of 50,000 of donor cells. After 16 h of overnight incubation, cells were either untreated (for physiological pH) or treated with trypsin or acidic pH, as described above. To distinguish between syncytial and intact cells, cells were fixed in ice-cold methanol for 10 min and then stained in 1% methylene blue in 23% ethanol and 0.008% KOH for 1 min and washed 3 times in tap water. Intact cells would be stained blue whereas syncytial cells would not take up the stain and appeared pink. Fusion at high cell densities triggered extensive syncytia formation resulting in cell death, cell lysis and complete disintegration of syncytia in the controls. Dead cells did not take up the blue dye and hence the controls would appear as an overall white/pale blue background. Inhibition in syncytia formation would reduce the extent of syncytia disintegration and restored the blue colour either by intact cells or by smaller syncytia. Therefore, the overall blue/white colour in a single field could be used as a marker for fusion inhibition, in addition to visual detection of intact cells and syncytia. Microscopic images were acquired using an Olympus CKX53 and Olympus EP50 camera and OSD software. Fusion index was calculated as (S-N)/T where S = number of nuclei in syncytia; N = number of syncytia; T = total number of nuclei.

### Pseudovirus infections

Production and infection of pseudoviruses were described in (Chan et al., 2021) with modifications. 293T-ACE2/293T-ACE2-TMPRSS2 cells seeded at 25,000 cells and Huh-7 cells seeded at 10,000 cells per well of 96-well plates were pre-treated with 10 μM of individual drugs, in duplicate, for 1 h. After 1 h, 50 μl of pseudovirus were added together with drugs to maintain the final drug concentration at 10 μM. Positive controls of pseudovirus treated with solvents (DMSO or water) and a negative control of bald pseudovirus (empty vector) were included. A no cell control treated with DMSO/water was used as background control. After incubation at 37°C/5%CO_2_ for 48h, they were tested for luciferase activity for % infectivity relative to the infected, solvent controls and for % viability relative to the un-infected solvent controls using XTT assay.

### Enzyme-linked immunosorbert assay

A 96-well ELISA plate was coated with 500 ng of recombinant ACE2 protein (BIOSS) per well in a humidifying chamber at 4°C overnight. After washing once with PBS, blocked in 3% BSA at 37°C for 2 h and a further wash in PBS, the ACE2-coated wells were pre-incubated with 31 μg/ml neutralizing antibody (NR-53796, BEI Resources), 10 μM drug or solvent control in 0.05% Tween 20/PBS at 37°C for 1 h. 500 ng of spike protein (NR-53937, BEI Resources) in 0.05% Tween 20/PBS were added to each well in the presence of respective neutralizing antibody, drug or solvent at 37°C for 2 h. After washing 3x in 0.05% Tween 20/PBS, 1:250 anti-spike antibody (NR-52947; BEI Resources) in 0.05% Tween 20/PBS was added to each well to incubate at 37°C for 1 h. After washing 3x in 0.05% Tween 20/PBS, 1:1,000 horseradish peroxidase (HRP)-conjugated anti-rabbit antibody (Cell Signaling) in 0.05% Tween 20/PBS was added to each well to incubate at 37°C for 1 h. The wells were washed 3x with 0.05% Tween 20/PBS and 3x with PBS. Colour was developed by incubating with 0.4 mg/ml o-phenylenediamine dihydrochloride substrate (Sigma) in 0.05 M Na_2_HPO4, 0.024 M citric acid, pH5 and 0.012% H_2_O_2_ for 30min at room temperature in the dark and read at 450 nm using a microplate reader (Bio-Tek Synergy HT).

### Western blotting

Western blotting was performed as described (MacCallum et al., 2006; Chan, 2016; Mufrrih et al., 2021). Protein lysates were harvested into 2xSDS-PAGE loading buffer (0.125 M Tris pH6.8, 4% SDS, 10% β-mercaptoethanol, 20% glycerol, 0.004% bromophenol blue). Proteins from equal number of cells together with recombinant ACE2 protein (BIOSS) or spike protein (BEI Resources NR-53937) standards were separated on TGX Stain-Free SDS-PAGE gel (Bio-Rad), transferred to polyvinylidene difluoride membrnaes (Millipore), blocked in 5% semi-skimmed milk (Marvel) in 0.1% Tween 20 (Sigma)/TBS (50 mM Tris pH 7.4, 150 mM NaCl) before being probed against primary and HRP-conjugated secondary antibodies in blocking buffer. Anti-ACE2 antibody (Proteintech) was used at 1:2000 and anti-mouse HRP (Cell Signaling Technology) at 1:1,000. Anti-SARS-CoV-2 spike antibody (BEI Resources NR-52947) was used at 1:1,000 and anti-rabbit HRP (Cell Signaling Technology) at 1:1,000. Protein bands were detected using Clarity™ ECL substrate (Bio-Rad). Images were captured and quantified using ChemiDoc™ XRS + system (Bio-Rad) and ImageLab 6.0.1 software (Bio-Rad).

### Statistical analysis

Statistical analysis was performed and graphs were plotted using Prism 9.3.1 (GraphPad). Ratio paired *t*-test was used to compare data between various viral envelope proteins and the empty vector control. Shapiro-Wilk normality test and one sample *t*-test were used for the analysis of drug fusion inhibition and negative screen against a theoretical mean of 1 and viability data against a theoretical mean of 100. A *p* value of <0.05 was considered statistically significant.

## Results

### Rationale for the fusion assays

SARS-CoV-2 enters cells by the plasma membrane and endosomal fusion pathways (Hoffmann et al., 2020; Ou et al., 2021). We, therefore, used the 293T-ACE2-TMPRSS2 cell line to represent canonical TMPRSS2-triggered plasma membrane fusion. In the case of SARS-CoV, fusion can be activated by exogenous trypsin, thermolysin, elastase, human airway trypsin-like protease and Factor Xa, thus may represent alternative fusion pathways (Simmons et al., 2004; Matsuyama et al., 2005; Du et al., 2007; Bertram et al., 2011). Trypsin has also been found to activate SARS-CoV and SARS-CoV-2 infection and fusion (Letko et al., 2020; Xia et al., 2020; Kim et al., 2022). Trypsin cleaves at both the S1/S2 R667 and the S2′ R797 of the SARS-1-S (Belouzard et al., 2009) and at multiple sites within the S1/S2 of the SARS-2-S (Jaimes et al., 2020; Mustafa et al., 2021). The S2’ site is conserved between SARS-CoV and SARS-CoV-2 (Bestle et al., 2020), therefore, we used trypsin to represent an alternative fusion model.

In the absence of TMPRSS2, SARS-CoV-2 enters cells by the cathepsin L-activated endosomal pathway (Ou et al., 2020). In the case of SARS-CoV, an acidic pH is not strictly required as long as the cathepsin L has been pre-activated (Simmons et al., 2005). This is in stark contrast to other viruses which require an acidic pH to induce conformational changes (White and Whittaker, 2016). With increasing discovery of canonical and non-canonical spike protein cleavage sites and the emerging roles of other proteases and matrix metalloproteases (MMPs) in SARS-CoV-2 infection (Essalmani et al., 2022; Fraser et al., 2022; Zhang et al., 2022; Zhao et al., 2022), it is possible that there are other unidentified fusion triggers apart from TMPRSS2 and cathepsin L cleavages. Emerging evidence suggests that SARS-CoV-2 requires an acidic pH for plasma membrane fusion (Kreutzberger et al., 2022) and (together with Ca^2+^) to cause conformation changes in fusion (Singh et al., 2022). Hence, we investigated pH5-triggered and pH5+trypsin-triggered fusion.

Several studies have detected fusion of ACE2(+) cells with the SARS-2-S at physiological pH in the absence of any exogenous triggers (Nguyen et al., 2020; Ou et al., 2020; Zhao et al., 2021b; Hörnich et al., 2021). It is thought that this fusion is required for syncytia formation but not entry (Nguyen et al., 2020) and is mediated by a MMP, most likely a disintegrin and metalloprotease 10 (ADAM10) which cleaves the spike protein at or near the S2’ site and near the S1/S2 site (Hörnich et al., 2021; Jocher et al., 2022; Yamamoto et al., 2022). Hence, we used this model to represent syncytia formation in TMPRSS2(-) cells.

Fusion assays are based on direct morphological criteria (Braga et al., 2021) or on reporter gene (luciferase, green fluorescent protein (GFP), β-galactosidase) complementation, either by using split genes (Buchrieser et al., 2021; Kandeel et al., 2021; Plaper et al., 2021; Rajah et al., 2021; Thakur et al., 2021; Zhang et al., 2021) or by segregation of transactivator and promoter (Zhao et al., 2021a; Hörnich et al., 2021). In this study, we opted for the phage T7 promoter-luciferase reporter gene complementation system because luciferase assay is rapid, objective, quantitative, and very sensitive with a wide linear range of at least 7 orders of magnitude (Supplementary Figure S1). Luciferase assay is highly accurate due to its ability to sample a large number of cells in 96-well, 24-well or 6-well formats, in contrast to GFP-microscopic method which is limited by the number of fields (hence number of cells) that the microscope can capture. Therefore, luciferase assay is an ideal choice for drug screening. One drawback of luciferase assay is that it cannot detect morphological changes, which will need to be supplemented by morphological fusion assay in downstream validation.

### Quantitative fusion assays for SARS-CoV-2

To study fusion in SARS-CoV-2, we developed a surrogate cell fusion assay. The fusion assay involved the segregation of a T7 promoter-driven luciferase reporter gene and the T7 polymerase in two cell types with one expressing the ACE2 protein (target cells) and the other one expressing the viral envelope protein (donor cell) (Figure 1). Fusion of the two cell types would enable the T7 polymerase to drive transcription of the reporter gene and fusion activity could be measured as a luciferase read-out. The β-galactosidase gene transcribed by the CMV promoter was used as an internal control. Western blotting confirmed expression of the spike protein in 293T-transfected cells and expression of the ACE2 in 293T-ACE2 and 293T-ACE2-TMPRSS2 cells (Supplementary Figure S2).

Fusion assays have been described for screening of inhibitors of fusion at physiological pH, using complementation (Zhao et al., 2021a; Thakur et al., 2021) or microscopic detection of syncytial sizes (Braga et al., 2021). We need a fusion assay that is optimized for drug screening and can be adapted to study a range of fusion triggers. In this study, we optimized the fusion conditions for drug screening e.g. transfection, co-culture seeding densities, length of co-culture, treatment conditions. We showed that we could control the level of syncytia formation by adjusting the seeding densities. Low co-culture densities were suitable for luciferase assay by limiting syncytia formation-induced cell death to achieve high luciferase read-outs (Supplementary Figure S3). Low and high co-culture densities were both suitable for morphological fusion assays. We found that high co-culture densities caused extensive syncytia formation, cell lysis and complete syncytia disintegration in the controls resulting in pale-coloured fields (Supplementary Figure S4). Fusion inhibitors could be easily identified by restoration of the blue colour without laborious cell counting. Next, we optimized the treatment conditions to 2 min to reduce the harmful effects of trypsin and acidic pH but still enabled the detection of sufficiently high luciferase read-outs to be used in screening (Supplementary Figure S5). This treatment protocol could be further adapted for other protease triggers e.g., cathepsin L, human airway trypsin-like protease.

As formation of syncytia would lead to cell death which might impact on the reporter gene activities, we performed a time-course experiment to evaluate optimal detection time for reporter gene activities and its variation with degree of fusion. The β-galactosidase activity remained constant and changes in fusion activity were mainly determined by the luciferase activity (Supplementary Figures S6, S7). Upon seeding of 293T-ACE2-TMPRSS2 at pH7.4, the co-culture remained as single cells until 2 h post-fusion (Supplementary Figures S6). However, a 5-fold increase in the fusion luciferase activity was detected at 1 h post-fusion before fusion was visible. At 2 h post-fusion, cells started to fuse to form small syncytia, which increased in size with time until extensive formation of big syncytia at 10 h post-fusion, concomitant with increases in fusion luciferase activity which peaked at 10 h or between 10 h-16 h post-fusion. Cells were not fully adhered until after an overnight incubation. At 16 h post-fusion, syncytia started to contract and pull away from substratum and rounded off to form giant balls until 24 h post-fusion when almost all cells had fused and contracted into giant balls. The fusion luciferase activity was sustained from 16 h to 20 h and slightly dropped at 24 h post-fusion. We chose 16 h or 20 h post-fusion as the detection time for fusion luciferase activity in 293T-ACE2-TMPRSS2 cells at physiological pH when cells were fully adhered and the fusion luciferase activity was still sustained. 293T-ACE2 cell fusion at physiological pH underwent a similar pattern of fusion but with a slower kinetics and much less extensive fusion. Fusion luciferase activity was detected from 2 h post-fusion and remained 2-4-fold lower than that of 293T-ACE2-TMPRSS2. Morphologically, syncytia formation were localized and appeared sporadically amongst single cells and was never complete even at 24 h post-fusion. Similarly, we chose 16 h or 20 h post-fusion as the detection time for fusion luciferase activity in 293T-ACE2 cells at physiological pH. For fusion at pH 7 + trypsin, pH 5 and pH 5 + trypsin, cells were treated for 2 min after a 16 h co-culture of 293T-ACE2 cells with donor cells at a slightly alkaline medium (pH∼8.3) to suppress pre-treatment fusion. Only localized and sporadic small syncytia formation was present before treatment (Supplementary Figure S7). For all three conditions, the kinetics of syncytia formation was fast which increased with time, concomitant with stable fusion luciferase activity until 5 h post-fusion, showing that pH7 + trypsin, pH5 and pH5 + trypsin are fusion triggers. In trypsin-treated cells, the syncytia started to contract and pull away from substratum and rounded off into giant cells from 6 h post-fusion, accompanied by a drop in fusion luciferase activity. In pH5-treated cells, syncytia formation was more localized and sporadic until 5 h post-fusion and became non-viable after 6 h post-fusion, concomitant with a drop in fusion luciferase activity. Therefore, we chose 5 h post treatment as the detection time for fusion luciferase activity.

**FIGURE 2:**
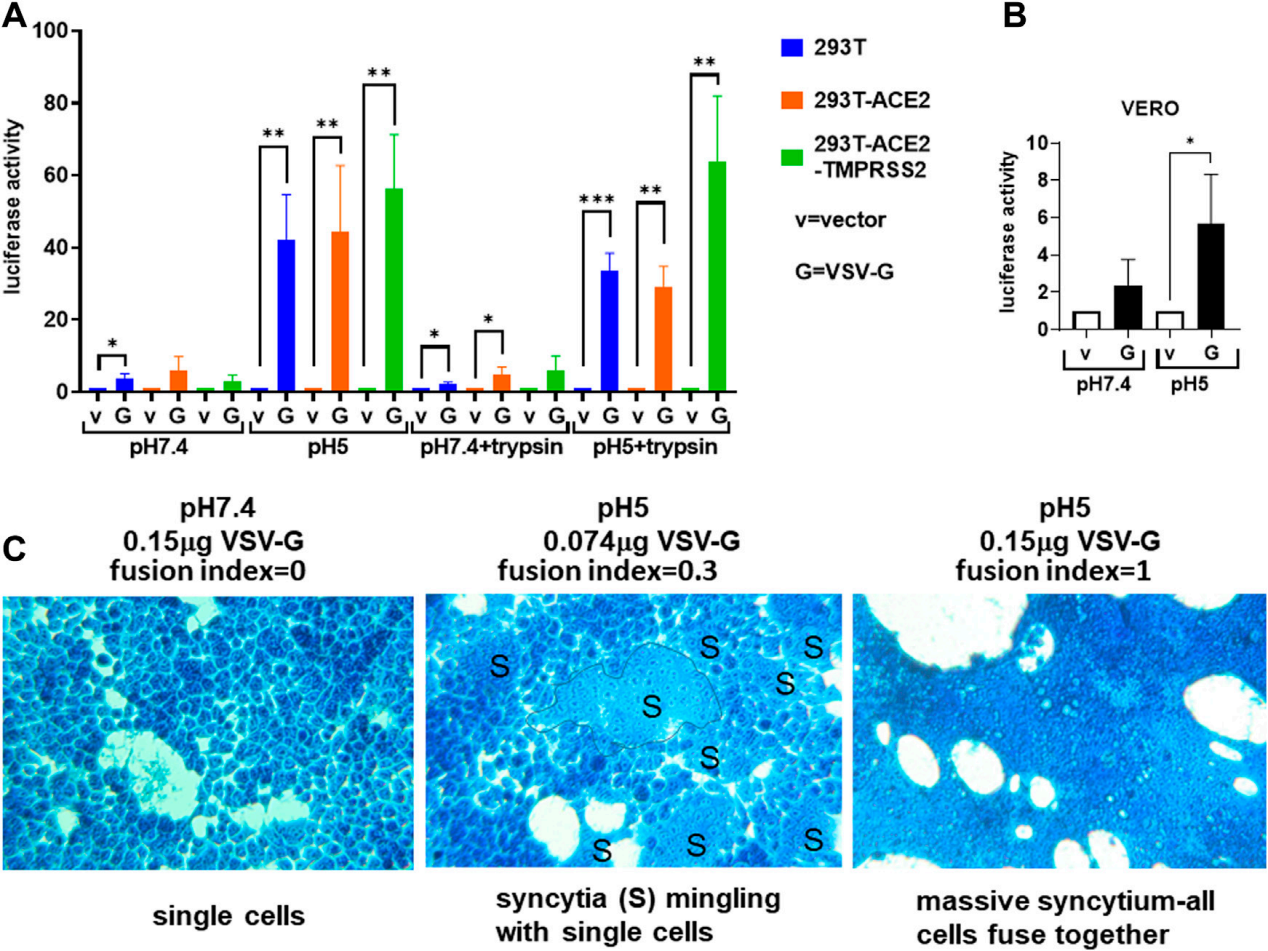
Vesicular stomatitis virus glycoprotein triggers universal acidic pH fusion. **(A,B)** Donor 293T cells were transfected with empty vector (negative control) or the vesicular stomatitis glycoprotein (VSV-G) together with a plasmid encoding the T7 polymerase. 19.000 donor cells were co-cultured with 19,000 target cells **(A)** (293T, 293T-ACE2, 293T-ACE2-TMPRSS2) or **(B)** Vero cells transfected with the luciferase and β-galactosidase reporter genes. The co-cultures were treated as indicated. Fusion activity was measured as luciferase activity normalized against β-galactosidase activity and expressed as a ratio to their respective vector control in the respective cell type and fusion condition. Data are presented as mean +/− SD of 3 repeats. *p < 0.05, **p < 0.01, and ***p < 0.001. **(C)** Donor 293T cells transfected with 0.15 μg of VSV-G (left and right) or 0.074 μg of VSV-G (middle) were co-cultured with target 293T cells and fusion triggered by incubating with pH7.4 (left) or pH5 (middle and right) fusion buffers. Cells were fixed and stained with methylene blue. A syncytium was outlined. Fusion index was calculated as (S–N)/T where S = number of nuclei in syncytia; N = number of syncytia; T = total number of nuclei. All photomicrographs are of the same magnification and scale.

We confirmed the specificity of the fusion test using the VSV-G as a control, which has been known to induce acidic but not physiological pH fusion, irrespective of cell types (Regan and Whittaker, 2013). We confirmed that the VSV-G mediated a much higher level of fusion luciferase activity at pH 5 compared to that at pH 7.4, with and without trypsin, in all cell types tested: 293T, 293T-ACE2, 293T-ACE2-TMPRSS2 cells and Vero cells (Figures 2A,B). There was little to no fusion in cells in the absence of the G protein (i.e. empty vector control). Microscopically, fusion was only detected at pH 5 (Figure 2C). 293T cells transfected with the VSV-G protein remained as single cells at physiological pH but readily formed syncytia when the pH was switched to 5 (Figure 2C). Specificity of the fusion assay was demonstrated by VSV-G dose-dependent syncytia formation in 293T cells. 293T cells transiently transfected with 0.15 μg of the VSV-G plasmid DNA formed extensive syncytia than those transfected with 0.074 μg of the VSV-G plasmid DNA (Figure 2C).

**FIGURE 3:**
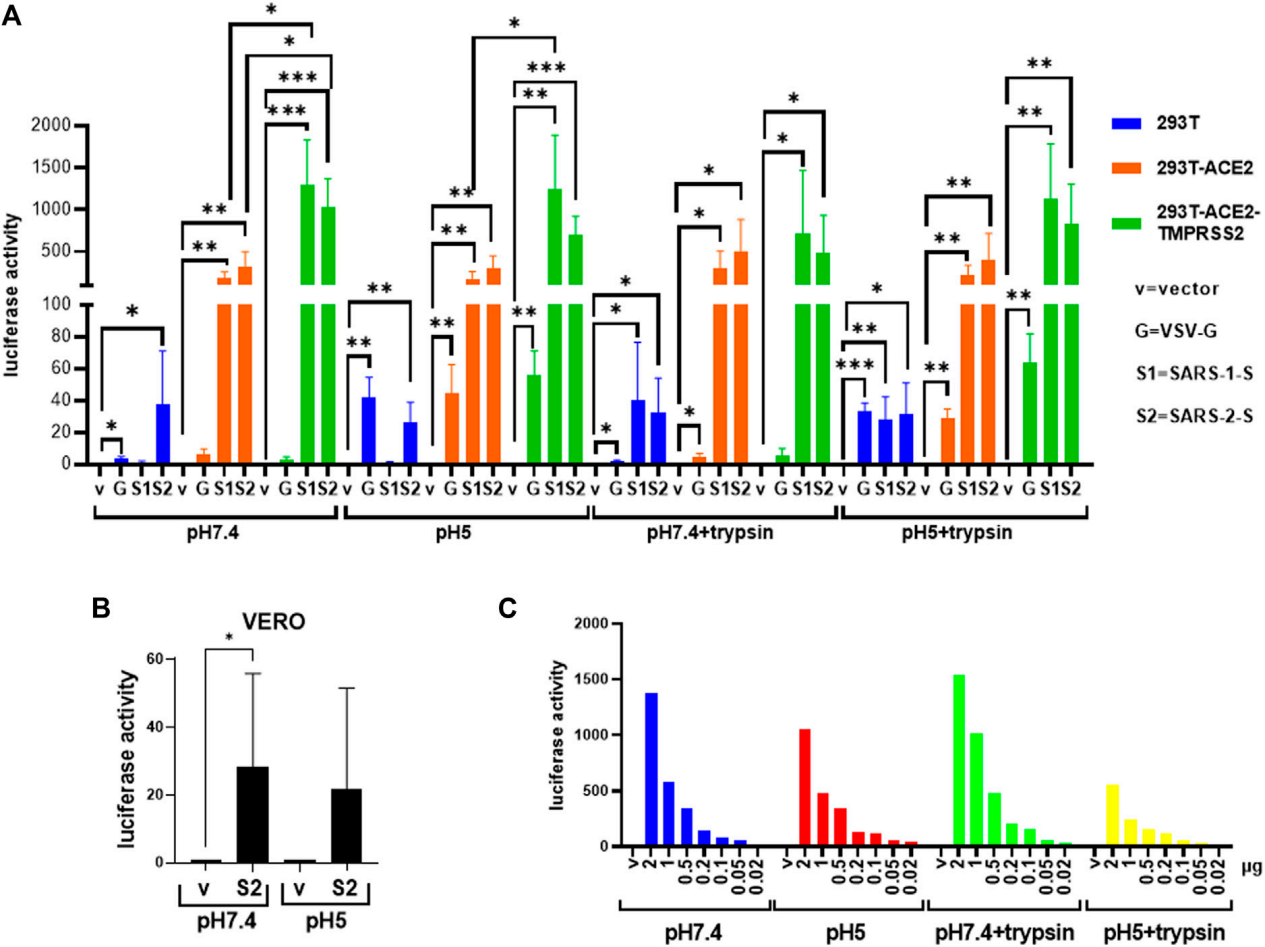
Severe acute respiratory syndrome coronavirus 2 spike protein triggers fusion in different cell types and fusion conditions. **(A,B)** Donor 293T cells were transfected with empty vector (negative control) or the severe acute respiratory syndrome coronavirus 1 and 2 spike proteins (SARS-1-S, SARS-2-S), respectively together with a plasmid encoding the T7 polymerase. 19,000 donor cells were co-cultured with 19,000 target cells **(A)** (293T, 293T-ACE2, 293T-ACE2-TMPRSS2) or **(B)** Vero cells transfected with the luciferase and β-galactosidase reporter genes. The co-cultures were treated as indicated. Fusion activity was measured as luciferase activity normalized against β-galactosidase activity and expressed as a ratio to their respective vector control in the respective cell type and fusion condition. Data on the vesicular stomatitis virus glycoprotein (VSV-G) from Figure 2A are included for comparison purpose. Data are presented as mean +/− SD of 3 repeats. **(C)** Donor 293T cells transfected with an empty vector or serial doses of the plasmid expressing the SARS-2-S, as indicated, were co-cultured with target 293T-ACE2 cells under various fusion condition (*n* = 1). *p < 0.05, **p < 0.01, ***p < 0.001 and ****p < 0.0001.

**FIGURE 4:**
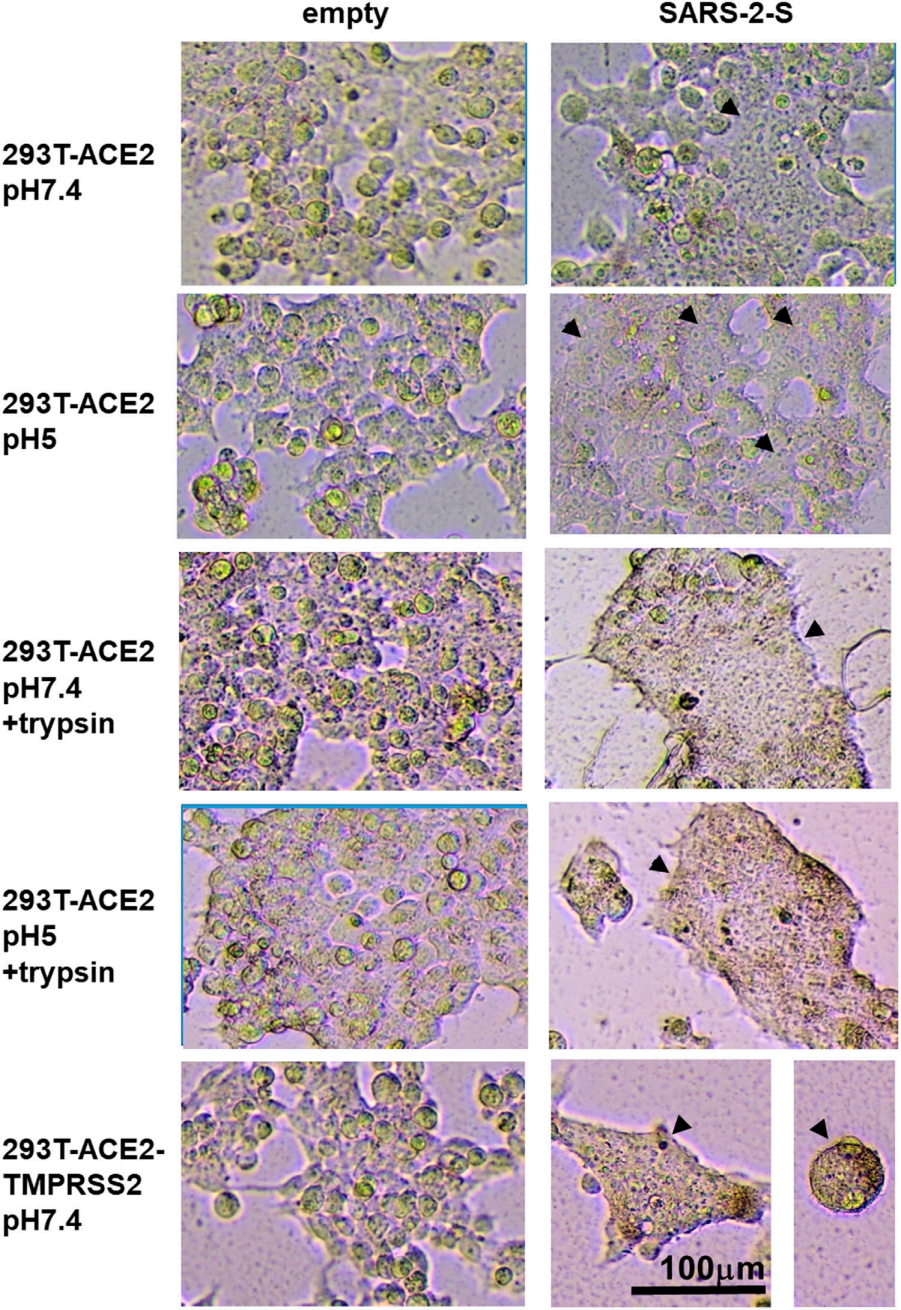
Syncytia formation is enhanced by TMPRSS2 or trypsin. 19,000 target cells (293T-ACE2, 293T-ACE2-TMPRSS2) transfected with the luciferase and β-galactosidase reporter genes were co-cultured with 19,000 donor 293T cells transfected with an empty vector or the severe acute respiratory syndrome coronavirus 2 spike protein (SARS-2-S) together with a plasmid encoding the T7 polymerase. The co-cultures were treated as indicated. Photomicrographs illustrate intact single cells and syncytia (arrowheads). Bright-field images are of the same magnification x100 and scale and from the same repeat.

In contrast, SARS-1-S and SARS-2-S only induced fusion in the presence of ACE2 either ectopically expressed in 293T-ACE2 and 293T-ACE2-TMPRSS2 cells or endogenously expressed in Vero cells, confirming the requirement of ACE2 in SARS-CoV and SARS-CoV-2 fusion (Figures 3A,B, Figure 4, Supplementary Figures S8–S11). There was little to no background fusion of cells in the absence of ACE2 (i.e., 293T cells) or spike protein (i.e., empty vector control). SARS-2-S, but not SARS-1-S, induced background levels of fusion luciferase activity at pH 7.4 and pH 5 in 293T cells (Figure 3A). This could be due to recognition of alternative receptors (Cantuti-Castelvetri et al., 2020; Daly et al., 2020). More likely, this just reflects the sensitivity of the assays, as a similar background level of fusion luciferase activity was detected with the VSV-G fusion protein at non-permissive pH (pH 7.4). Trypsin induced background levels of fusion luciferase activity in ACE2(-) 293T cells, regardless of the nature of the fusion protein and pH permissivity, suggesting that trypsin alone induces a low level of non-specific fusion luciferase activity. TMPRSS2 is not strictly required for fusion as fusion occurred in TMPRSS2(-) 293T-ACE2 and Vero cells although the presence of TMPRSS2 enhanced fusion mediated by both SARS-1-S and SARS-2-S at physiological pH and pH5. In the presence of trypsin, there was no significant difference between fusion luciferase activity in 293T-ACE2 and 293T-ACE2-TMPRSS2 cells, suggesting that trypsin can replace TMPRSS2 in mediating fusion in both SARS-CoV and SARS-CoV-2. Fusion luciferase activity was similar in TMPRSS2(-) 293T-ACE2 cells under all four conditions and was equally less efficient than that in TMPRSS2(+) cells, suggesting that TMPRSS2 confers an advantage to the SARS-CoV-2 in fusion (and hence infection). Serial dilutions of the SARS-2-S could not distinguish between fusion efficiency under different conditions in 293T-ACE2 cells (Figure 3C). Microscopically, the 293T-ACE2-TMPRSS2 cells fused readily at physiological pH (Figure 4, Supplementary Figure S8). All cells were fused into giant syncytia. Extensive fusion resulted in syncytia pulling away from substratum and condensed into giant balls. Trypsin induced extensive fusion of the 293T-ACE2 cells into big syncytia at both pH 7.4 and pH 5 whereas pH 7.4 and pH 5 alone only induced sporadic syncytia scattered amongst single cells and the syncytia were much smaller in size (Supplementary Figure S8–S11). In concordance with results from other groups (Ou et al., 2020; Xia et al., 2020; Buchrieser et al., 2021; Hörnich et al., 2021), our results indicate that syncytia formation can take place in the absence of TMPRSS2 and trypsin but is most efficient in their presence. In addition, we demonstrated a pH5-triggered fusion process.

#### Inhibition profiles confirm specificity of fusion assays

**FIGURE 5:**
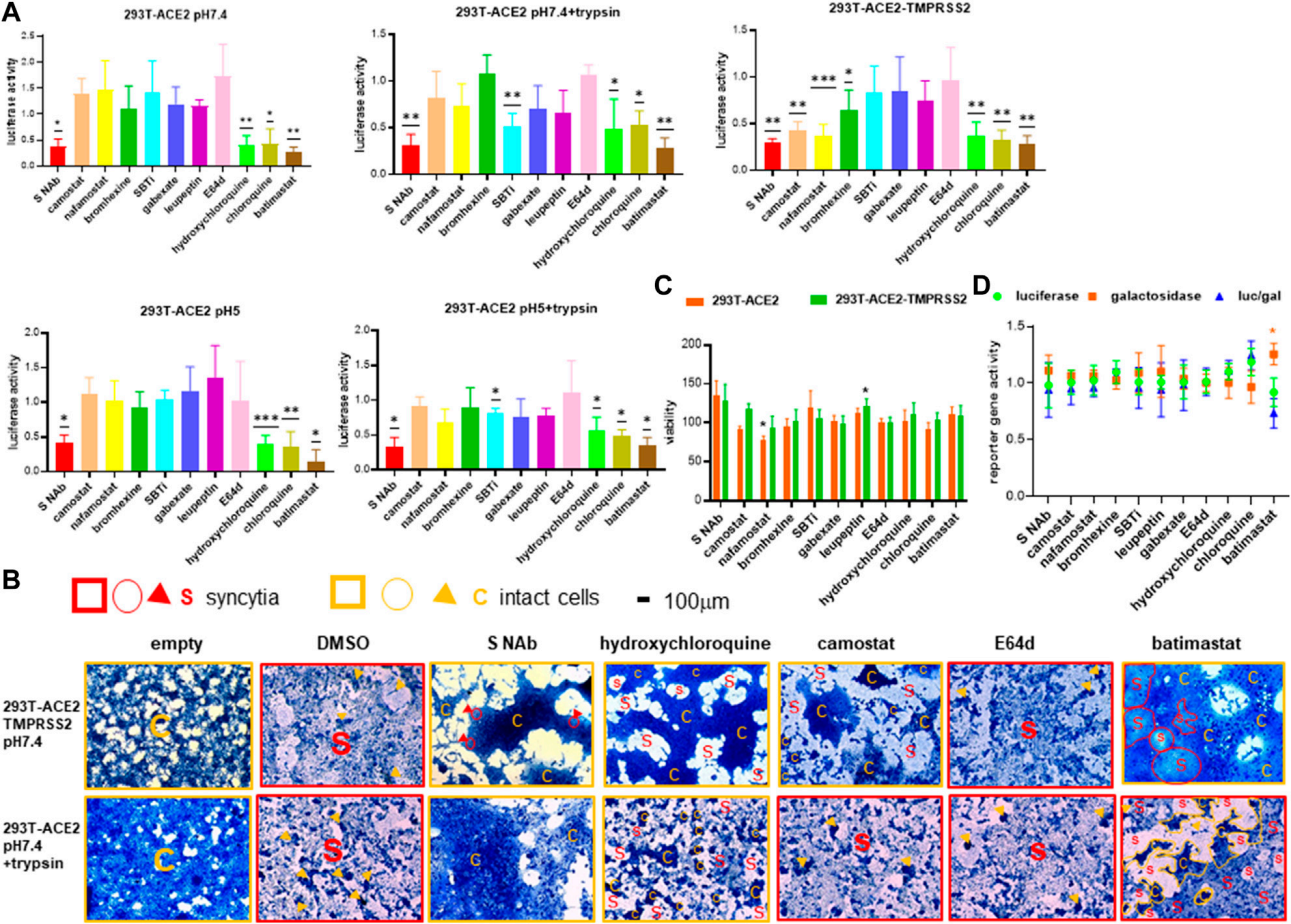
Specificity of different modes of fusion. **(A)** 19,000 target cells (293T-ACE2, 293T-ACE2-TMPRSS2) transfected with the luciferase and β-galactosidase reporter genes were pre-incubated with 10 μg/ml of anti-spike monoclonal antibody (ACROB SPD-M128), 75 μg/ml soybean trypsin inhibitor (SBTi), 10 μg/ml leupeptin or 10 μM of individual drugs before co-cultured with 19,000 donor 293T cells transfected with the severe acute respiratory syndrome coronavirus 2 (SARS-CoV-2) spike proteins (SARS-2-S) together with a plasmid encoding the T7 polymerase. The co-cultures were treated as indicated. Fusion activity was measured as luciferase activity normalized against β-galactosidase activity and expressed as a ratio to the solvent control in the respective cell type and fusion condition which is set as 1. Data are presented as mean+/-SD of >3 repeats. **(B)** Photomicrographs of fusion cell morphology. 50,000 target cells (293T-ACE2, 293T-ACE2-TMPRSS2) pre-incubated with 28 μg/ml anti-spike monoclonal antibody (Sino Biol, 40592-R001) or 10 μM of individual drugs were co-cultured with 50,000 donor 293T cells transfected with an empty vector or the SARS-CoV-2 spike protein. The co-cultures were treated as indicated. Cells were fixed and stained with methylene blue. Bright-field images are of the same magnification x100 and scale. Fields of entire or mostly syncytia formation are framed with red squares. Fields of entire or mostly intact cells are framed with orange squares. Scattered syncytia are circled or outlined red and labelled S. Scattered intact cells are circled or outlined orange and labelled C. Sporadic intact cells and syncytia are marked by orange and red arrowheads, respectively. **(C)** Viability was measured by XTT assays in fusion cells (293T + 293T-ACE2 or 293T + 293T-ACE2-TMPRSS2) under physiological pH and treated with compounds as in **(A)**. Data are expressed as % viability to solvent control which is set as 100%. Data are presented as mean+/-SD of three repeats. **(D)** Negative screen. One of the three cell types: 293T, 293T-ACE2 and 293T-ACE2-TMPRSS2 (30,000 cells) was co-transfected with plasmids encoding the T7 polymerase, luciferase and β-galactosidase reporter genes and then treated with compounds as in **(A)**. Luciferase, β-galactosidase and the luciferase/β-galactosidase ratio are presented as ratios to the solvent controls which are set as 1. Data are presented as mean+/-SD of three repeats *p < 0.05, **p < 0.01, and ***p < 0.001.

To further confirm the specificity of the SARS-CoV-2 fusion assays, we established inhibition profiles using a number of known proteases and inhibitors. Using two independent monoclonal neutralizing antibodies targeting the SARS-2-S RBD in the reporter gene fusion assay and morphological fusion assay, respectively, we confirmed that our fusion assays are specific for the SARS-2-S (Figures 5A,B). Fusion in the 293T-ACE2-TMPRSS2 cells was significantly inhibited by the specific TMPRSS2 inhibitors, camostat and nafamostat and less potently by the mycolytic agent, bromhexine, but not by other generic trypsin (SBTi), serine (gabexate) and serine, threonine, cysteine protease (leupeptin) inhibitors and the cathepsin and calpain inhibitor, E64d. Camostat, nafamostat and bromhexine did not inhibit fusion in 293T-ACE2 cells under physiological and acidic pH with and without trypsin treatment, confirming the requirement of TMPRSS2 in fusion in TMPRSS2(+) cells but not in TMPRSS2(-) cells. Altogether these results confirm a TMPRSS2-specific mechanism of fusion in TMPRSS2(+) cells. Trypsin-induced fusion in the 293T-ACE2 cells was significantly inhibited by SBTi at both pH 7.4 and pH 5, but not by other specific or generic proteases (Figure 5A). Inhibition by SBTi was specific to trypsin-induced fusion in the 293T-ACE2 cells as it did not inhibit fusion mediated by pH 7.4 and pH 5 in the 293T-ACE2 or in the 293T-ACE2-TMPRSS2 cells. Altogether these results confirm a pH-independent trypsin-triggered fusion mechanism in the 293T-ACE2 cells. Fusion of 293T-ACE2 cells at pH 7.4 and pH 5 was not inhibited by any of the above serine, threonine, cysteine proteases or trypsin inhibitors, thus representing another fusion model. In addition, fusion under all conditions and cell types, was potently inhibited by hydroxychloroquine, chloroquine and batimastat; suggesting that they block a common pathway that is independent of TMPRSS2, pH or trypsin.

To exclude the possibility that the reduced luciferase activity was a result of cytotoxicity, we used an XTT viability assay to confirm that the neutralizing antibody/drugs were non-cytotoxic apart from nafamostat, which reduced 293T-ACE2 cell viability to 75% (Figure 5C). To exclude the possibility that the reduced luciferase activity was a result of inhibition of the T7 promoter driving the transcription of the luciferase reporter gene instead of fusion, we performed a negative screen by co-transfection of the 293T cells with the CMVT7; T7EMCVluc and CMVβ-gal plasmid DNAs. We confirmed that none of the neutralizing antibody/drugs suppressed T7 or CMV promoter activity (Figure 5D).

Overall, we have confirmed the specificity of the fusion assays to differentiate between TMPRSS2-dependent plasma membrane fusion, TMPRSS2-independent trypsin-triggered fusion and syncytia formation at physiological pH.

### Inhibition profiles of top drug hits reveal distinctive fusion triggers

**FIGURE 6:**
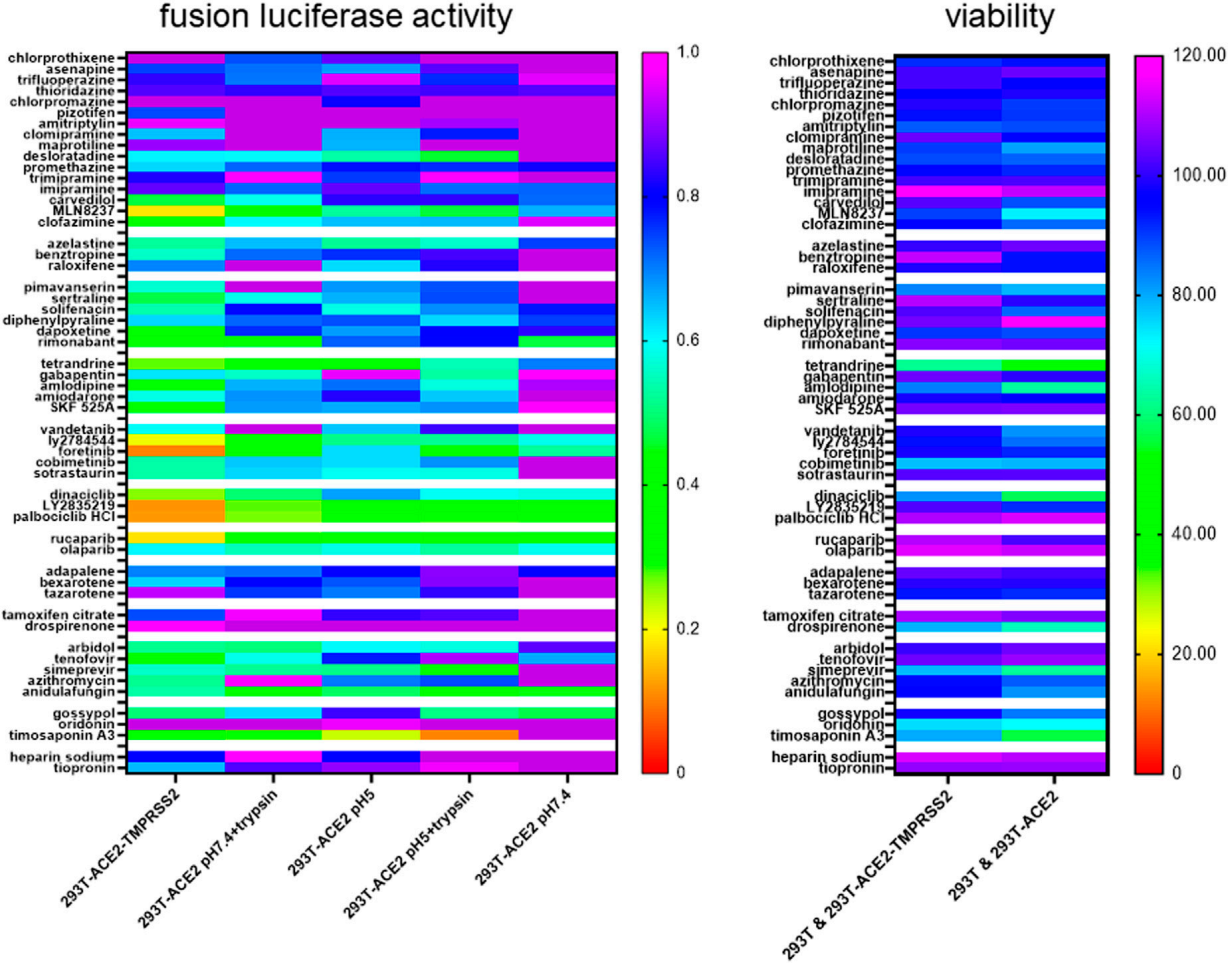
Heatmap of drug inhibition profiles of different modes of fusion. 19,000 target cells (293T-ACE2, 293T-ACE2-TMPRSS2) transfected with the luciferase and β-galactosidase reporter genes were pre-incubated with 10 μM of individual drugs before co-cultured with 19,000 donor 293T cells transfected with the severe acute respiratory syndrome coronavirus 2 (SARS-CoV-2) spike proteins together with a plasmid encoding the T7 polymerase. The co-cultures were treated as indicated. Fusion activity was measured as luciferase activity normalized against β-galactosidase activity and expressed as a ratio to the solvent control in the respective cell type and fusion condition which is set as 1. Data are presented as mean of 3 repeats. Viability was measured by XTT assays in fusion cells (293T + 293T-ACE2 or 293T + 293T-ACE2-TMPRSS2) under physiological pH and expressed as % viability to solvent control which is set as 100%. Data are presented as mean of three repeats. Data outside the range are depicted as dark purple.

Using pseudovirus particles, we recently identified small molecules that inhibited SARS-CoV-2 infection (Chan et al., 2021). To study whether these top hits inhibited SARS-CoV-2 infection at the fusion step, we screened select top hits for inhibition of ACE2(+)TMPRSS2(+) and ACE2(+)TMPRSS2(-) cell fusion under the above conditions. We divided the drug hits into different classes according to their structure and pharmacology and ranked them in order of inhibitory activity of SARS-CoV-2 infection using data from our previous study (Chan et al., 2021). Despite being identified using TMPRSS2(-) cells, a larger proportion of the drug hits inhibited fusion in 293T-ACE2-TMPRSS2 cells than in 293T-ACE2 cells (Figure 6, Supplementary Figure S12). 293T-ACE2 cell fusion at physiological pH was refractory to inhibition by most of our top drug hits. Moreover, the drug hits inhibited fusion in ACE2(+)TMPRSS2(+) cells more potently than in ACE2(+)TMPRSS2(-) cells and were the least inhibitory for fusion under physiological pH in ACE2(+)TMPRSS2(-) cells. In 293T-ACE2 cells, syncytial fusion at pH 7.4 displayed a different inhibition pattern to that of fusion at pH 5, pH 7 + trypsin, pH 5 + trypsin, further confirming the difference between syncytial fusion and entry fusion. Surprisingly, fusion at pH 5 shared an overall inhibition pattern with trypsin-induced fusion but not with fusion at physiological pH, suggesting that SARS-CoV-2 fusion may be activated directly by an acidic pH. Trypsin + pH 5-induced fusion shared a similar inhibition profile with that of pH 5 and pH 7 + trypsin but also displayed unique sensitivity to several kite-shaped molecules, i.e., desloratadine, promethazine, imipramine, suggesting that pH 5 + trypsin-induced fusion may represent a distinctive type of fusion. Overall, inhibition profiles of our top drug hits reveal distinctive fusion triggers and distinguish between TMPRSS2- and trypsin-triggered fusion, pH 7 + trypsin- and pH 5 + trypsin-triggered fusion, pH 5- and pH 5 + trypisn-triggered fusion.

Using XTT viability assays, we examined cytotoxicity of these drug hits during fusion of 293T with 293T-ACE2 or with 293T-ACE2-TMPRSS2. Overall, ACE2(+)TMPRSS2(+) fusion cells were more resistant to the toxic effects of the drugs (Figure 6). In 293T-ACE2 cells, tetrandrine, amlodipine, dinaciclib, drospirenone, simeprevir, oridonin and timosaponin A3 were very cytotoxic (51–72% viability), maprotiline, MLN8237, pimavanserin, vandetanib, cobimetinib, anidulafungin and gossypol were cytotoxic (73–84% viability), whereas chlorpromazine, amitriptylin, desloratadine, carvedilol, clofazimine, solifenacin, LY2784544 and azithromycin were slightly cytotoxic (85–90% viability).

**FIGURE 7:**
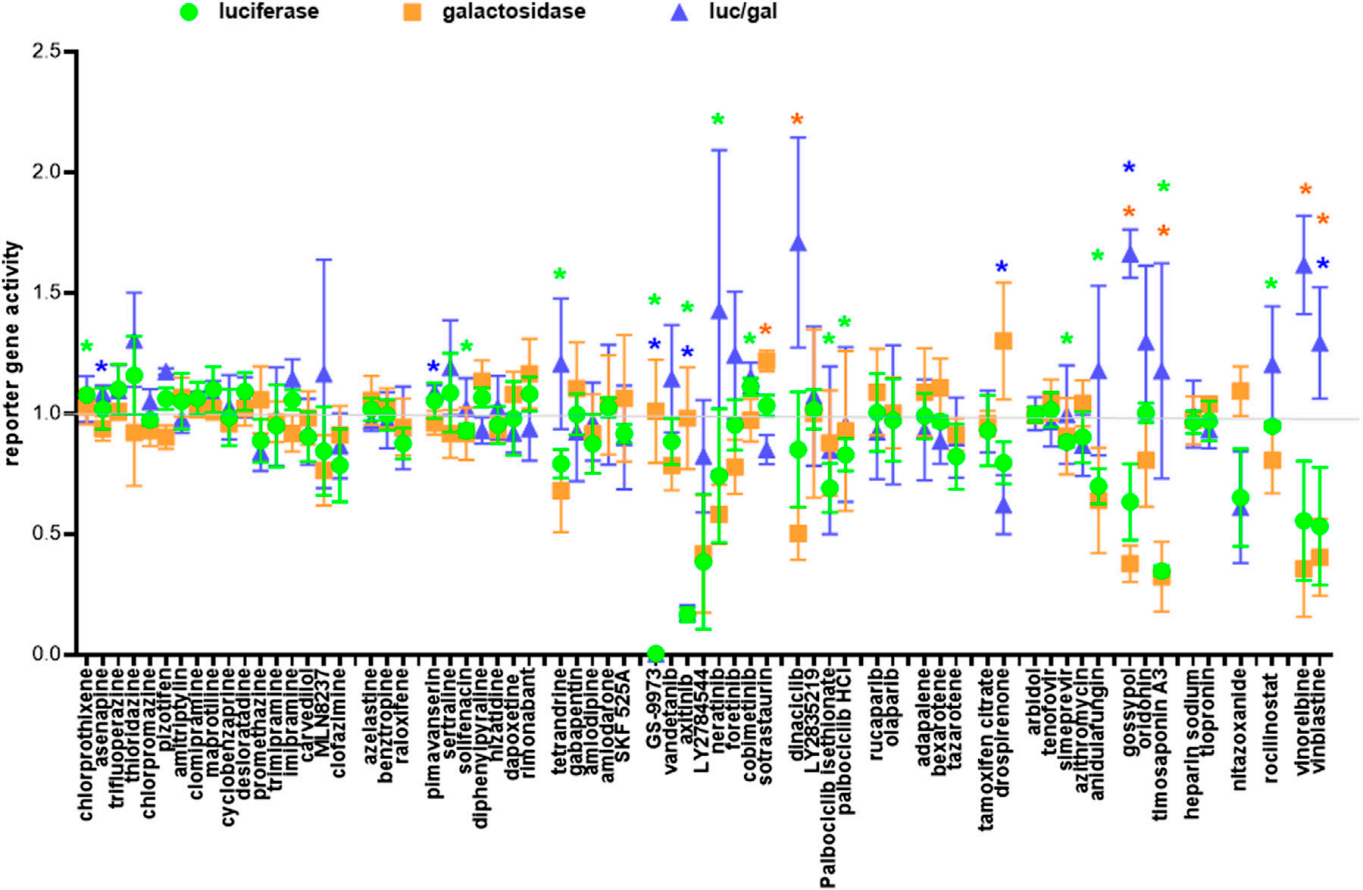
Negative screen. One of the three cell types: 293T, 293T-ACE2 and 293T-ACE2-TMPRSS2 (30,000 cells) was co-transfected with plasmids encoding the T7 polymerase, luciferase and β-galactosidase reporter genes and then treated with 10 μM of individual drugs. Luciferase, β-galactosidase and the luciferase/β-galactosidase ratio are presented as ratios to the solvent controls which are set as 1. Data are presented as mean +/− SD of three repeats. *p < 0.05

To exclude the possibility that the fusion hits inhibited the T7 promoter driving the transcription of the luciferase reporter gene instead of fusion *per se*, we performed a negative screen by co-transfection of 293T cells with the CMVT7; T7EMCVluc and CMVβgal plasmids before adding individual drug hits. Because fusion occurred between two cells, we performed the three repeats in the three cell types to avoid bias towards a single cell type. We identified true suppressor of the T7 promoter (reduced T7 promoter activity; unchanged CMV promoter activity; reduced luciferase/galactosidase ratio) i.e., GS-9973, axitinib, drospirenone, nitazoxanide (Figure 7). Molecules that simultaneously reduced the T7 and CMV promoter activity were implicated as cytotoxic and were not specific T7 promoter suppressor (luciferase/galactosidase ratio ≥1) i.e. tetrandrine, LY2784544, neratinib, dinaciclib, palbociclib isethionate, palbociclib HCl, anidulafungin, gossypol, timosaponin A3, vinorelbine, vinblastine. The rest of the fusion drug hits did not show significant suppressive effects on either the T7 or the CMV promoters.

### Lopsided kite-shaped molecules are potent fusion inhibitors

We have previously identified a class of kite-shaped molecules that inhibited pseudovirus infection at the entry/post-attachment step; however, the exact mechanism is undetermined (Chan et al., 2021). The entry/post-attachment step involves a number of steps including virus-host receptor binding, endocytosis, trafficking and membrane fusion. Our fusion assays would allow us to distinguish between steps that are directly involved in the fusion process (e.g., modulation of ACE2 or spike surface levels, inhibition of the ACE2/spike interaction, modulation of membrane fluidity, fusion *per se* and those that are not (e.g., endocytosis, trafficking). Using the above fusion assays, we screened kite-shaped molecules together with kitelike-shaped molecules and non-kite nervous system-acting drugs for inhibition of pH-dependent/independent and protease-dependent/independent fusion. Canonical kite-shaped molecules did not show significant inhibition of fusion at pH 7.4 with and without trypsin nor at pH5 in 293T-ACE cells (Figure 6, Supplementary Figure S12). 293T-ACE2 cell fusion at pH 5+trypsin was mainly inhibited by the class of H1 receptor inhibitors (desloratadine, promethazine, imipramine). Fusion of 293T-ACE2-TMPRSS2 cells was also inhibited by the H1 receptor inhibitor, promethazine, and by the dopamine receptor inhibitors, asenapine and thioridazine. Generally, kite-shaped molecules did not suppress the T7 or CMV promoters (Figure 7) in the negative screen, were non-cytotoxic (Figure 6) and were moderate fusion inhibitors in both luciferase and morphological fusion assays (Figure 6, Supplementary Figures S12, S13). Altogether, these results suggest that kite-shaped molecules do not majorly act on the fusion process but exert their anti-viral properties *via* other processes such as endocytosis and trafficking.

**FIGURE 8:**
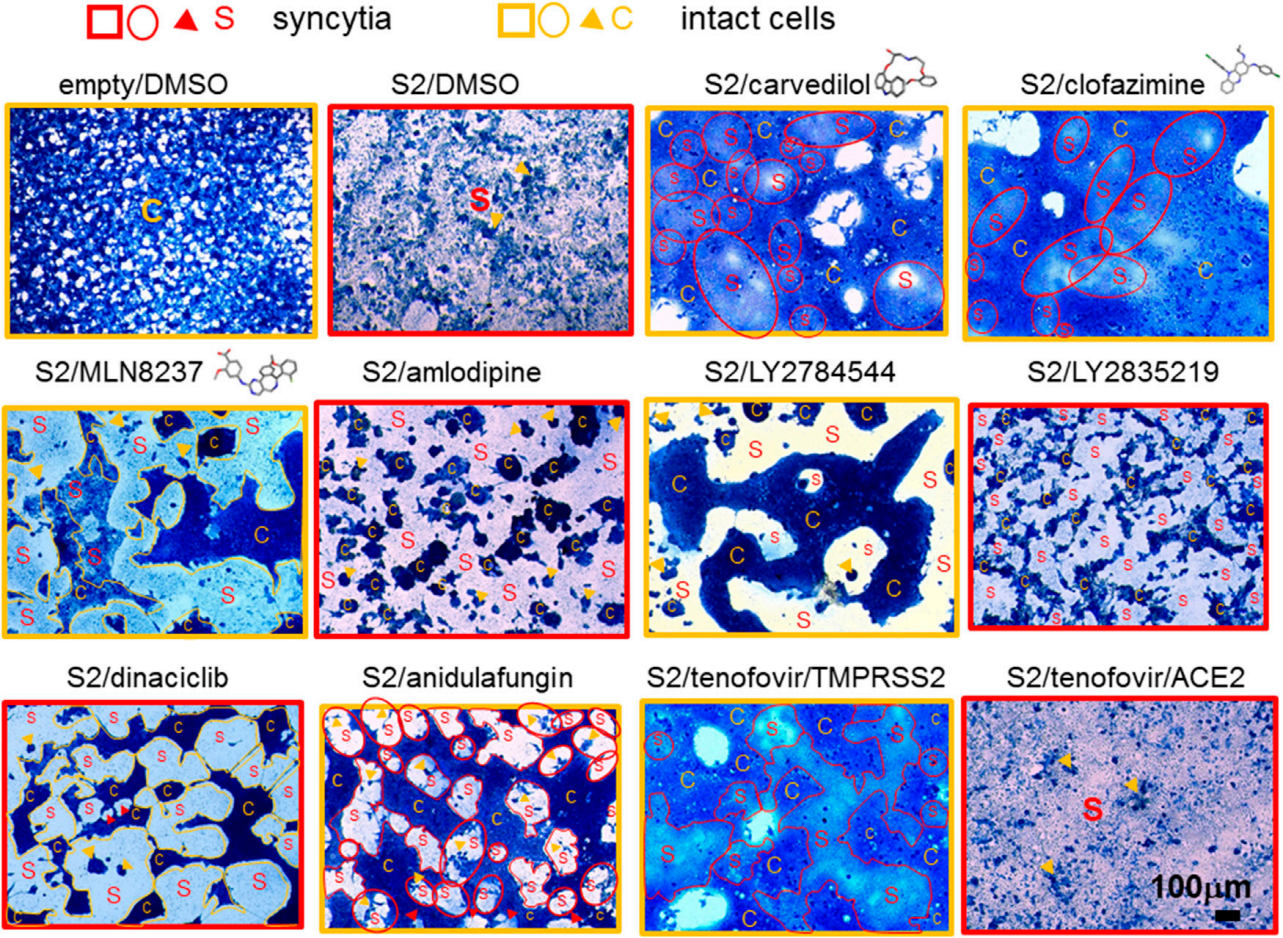
Fusion cell morphology. 50,000 293T-ACE2-TMPRSS2 pre-incubated with 10 μM of individual drugs were co-cultured with 50,000 donor 293T cells transfected with an empty vector or the SARS-CoV-2 spike protein (S2). 293T-ACE2 cells treated with tenofovir disoproxil fumarate under physiological pH was displayed at the bottom right corner; otherwise, all images represent fusion of 293T-ACE2-TMPRSS2 cells. Cells were fixed and stained with methylene blue. Bright-field images are of the same magnification x100 and scale. Fields of entire or mostly syncytia formation are framed with red squares. Fields of entire or moslty intact cells are framed with orange squares. Scattered syncytia are circled or outlined red and labelled S. Scattered intact cells are circled or outlined orange and labelled C. Sporadic intact cells and syncytia are marked by orange and red arrowheads, respectively. Drug images are from PubChem.

**FIGURE 9:**
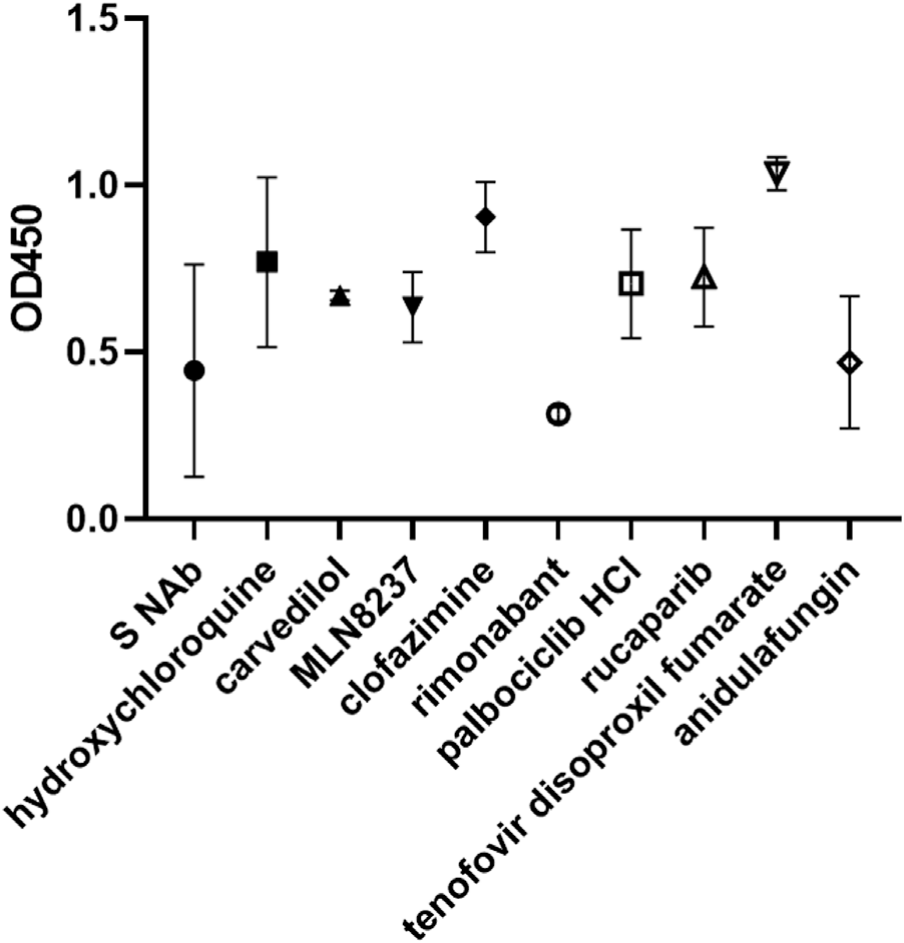
Rimonabant and anidulafungin inhibit ACE2-spike binding. Enzyme-linked immunosorbent assay of spike neutralizing antibody (S NAb) and drug inhibition of ACE2-spike binding. Data are presented as % binding of their respective solvent control and represent mean +/− SD of 2 repeats.

In contrast, lopsided kites were more potent fusion inhibitors. The lopsided kite, carvedilol, belonging to the class of adrenergic receptor inhibitors (Fisker et al., 2015), significantly inhibited fusion of 293T-ACE2 cells at pH 5 + trypsin (Figure 6, Supplementary Figure S12). Carvedilol was demonstrated to be a fusion inhibitor by inhibiting 63% of fusion luciferase activity and morphologically in 293T-ACE2-TMPRSS2 cells (Figures 6, 8, Supplementary Figure S12). Two other lopsided kites, clofazimine and MLN8237, belonging to different functional classes, exhibited potent inhibition of fusion luciferase activity. Clofazimine, is an anti-leprotic, anti-mycobaterial (Riccardi et al., 2020). It significantly inhibited fusion luciferase activity in 293T-ACE2-TMPRSS2 cells and at pH5+/-trypsin in 293T-ACE2 cells (Figure 6, Supplementary Figure S12). Its fusion inhibition was confirmed morphologically in 293T-ACE2-TMPRSS2 cells at high cell density (Figure 8). MLN8237 is an Aurora serine/threonine kinase inhibitor (Sankhe et al., 2021). It significantly inhibited fusion under all conditions apart from 293T-ACE2 cell fusion at physiological pH (Figure 6, Supplementary Figure S12). Its fusion inhibition was confirmed morphologically in 293T-ACE2-TMPRSS2 cells at high cell density (Figure 8). All three lopsided kites displayed some degree of cytotoxicity (Figure 6) but none of them was a T7 promoter suppressor in the negative screen (Figure 7). The near universal potency of MLN8237 prompted us to see whether it was due to inhibition of ACE2-spike binding. However, none of the lopsided kites inhibited ACE2-spike binding in an ELISA (Figure 9).

The kitelike-shaped azelastine, targeting the H1 receptor (Simons and Simons, 1999), significantly inhibited fusion under all conditions apart from 293T-ACE2 cell fusion at physiological pH (Figure 6, Supplementary Figure S12). Another kitelike-shaped benztropine, targeting the muscarinic and dopamine receptors (Reith et al., 2015), selectively inhibited fusion in 293T-ACE2-TMPRSS2 cells and pH7+trypsin-triggered fusion in 293T-ACE2 cells. In contrast, the kitelike-shaped raloxifene, targeting the oestrogen receptor (Yu et al., 2022), did not inhibit any of the fusion. These kitelike-shaped molecules did not suppress the T7 or CMV promoters (Figure 7) and were generally non-cytotoxic (Figure 6). Similar to the kite-shaped molecules, kitelike-shaped molecules were moderate inhibitors in both luciferase and morphological fusion assays (Figure 6, Supplementary Figures S12, S13).

Fusion in 293T-ACE2-TMPRSS2 cells was sensitive to inhibition by all non-kite nervous system-acting inhibitors targeting the serotogenic and muscarinic pathways and the cannabinoid CB1 receptor (Hegde, 2006; Oleson et al., 2012; Gillman and Gillman, 2019; Cignarella et al., 2021; Cummings et al., 2022) (Figure 6, Supplementary Figure S12). Fusion in 293T-ACE2 cells was sensitive to select non-kite nervous system-acting inhibitors under different conditions. Whereas 293T-ACE2 cell fusion at pH 7.4 was resistant to inhibition by most kite, kitelike and none-kite nervous system-acting drugs, it was significantly inhibited by rimonabant. Rimonabant also inhibited fusion in 293T-ACE2-TMPRSS2 cells and at pH7.4 + trypsin in 293T-ACE2 cells, suggesting that rimonabant may be a pH-dependent, trypsin-independent fusion inhibitor. However, rimonabant did not show a high level of morphological fusion inhibition at high cell density (Supplementary Figure S13), suggesting that rimonabant is a moderate fusion inhibitor. These non-kite nervous system-acting molecules did not suppress the T7 or CMV promoters (Figure 7) and were relatively non-cytotoxic (Figure 6). Similar to the kite-shaped molecules, the none-kite nervous system-acting molecules were moderate inhibitors in both luciferase and morphological fusion assays (Figure 6, Supplementary Figure S12, S13).

Overall, select kite-shaped and kitelike-shaped molecules and non-kite nervous system-acting drugs are moderate fusion inhibitors whereas lopsided kites are potent fusion inhibitors.

### Different modes of fusion display different calcium dependency

Ca^2+^ flux has been widely implicated in virus fusion (Millet and Whittaker, 2018). We have previously identified several drug hits with Ca^2+^-modulating ability that inhibited pseudovirus infection (Chan et al., 2021). In this study, we investigated their roles in virus fusion. None of these drugs inhibited fusion of 293T-ACE2 cells at pH7.4 (Figure 6, Supplementary Figure S12). Amiodarone, an inhibitor of the myocardial Ca^2+^, K^+^ and Na^+^ channels (Kodama et al., 1999), did not show significant inhibition of any of the fusion. Tetrandrine is a natural compound which blocks Ca^2+^ channel amongst its multi-inhibitory activity (Bhagya and Chandrashekar, 2022). It potently inhibited fusion luciferase activity in all other conditions (Figure 6, Supplementary Figure S12); however, this could be partly accounted for by its cytotoxicity (Figure 6). Indeed, tetrandrine’s cytotoxicity might explain its strong suppression of both the T7 and CMV promoters despite not being a specific T7 promoter suppressor (Figure 7). Morphologically, tetrandrine only inhibited a low level of fusion at high cell density (Supplementary Figure S13). Gabapentin is an analogue of the neurotransmitter, γ-aminobutyric acid, but it is also a Ca^2+^ channel blocker (Sills, 2006). It only showed significant, moderate inhibition of 293T-ACE2 cells at pH5+trypsin (Figure 6, Supplementary Figure S12). SKF 525A inhibits Ca^2+^ flux amongst its other inhibitory activity (Summers and Tillman, 1979). It showed moderate inhibition of 293T-ACE2 cell fusion at pH5 (+/-) trypsin and 293T-ACE2-TMPRSS2 cell fusion (Figure 6, Supplementary Figure S12, S13). The above compounds possess multi-functional activity. Perhaps more interesting was the Ca^2+^ channel blocker, amlodipine (Murdoch and Heel, 1991), which selectively inhibited luciferase and morphological fusion activity in 293T-ACE2-TMPRSS2 but not in 293T-ACE2 cells (Figures 6, 8, Supplementary Figure S12). Amlodipine was not a T7 promoter suppressor (Figure 7) but displayed a low level of cytotoxicity in 293T-ACE2-TMPRSS2 cells causing cell aggregation (Figures 6, 8). Altogether these results suggest different calcium dependence in different modes of fusion.

### Select kinase inhibitors inhibit fusion

Several kinases were found to inhibit SARS-CoV-2 pseudovirus infection in our previous study (Chan et al., 2021), therefore, we studied whether they inhibited fusion. In the negative screen the kinases turned out a number of true T7 promoter suppressor (reduced T7 promoter activity and unchanged CMV promoter activity) i.e., GS-9973, axitinib and cytotoxic molecules (reduced T7 and CMV promoter activity with a ∼1 luc/gal ratio) i.e., neratinib, LY2784544 (Figure 7). The tyrosine kinase inhibitor, vandetanib (Vozniak and Jacobs, 2012), a potent inhibitor of SARS-CoV-2 pseudovirus infection (Chan et al., 2021), did not inhibit fusion luciferase activity (Figure 6, Supplementary Figure S12). In contrast, two other tyrosine kinase inhibitors, LY2784544, foretinib, and an Aurora serine/threonine kinase inhibitor, MLN8237 (Huynh, 2010; Passamonti et al., 2012; Sankhe et al., 2021) (also a lopsided kite, see above), potently inhibited fusion luciferase activity under select conditions. Morphologically, LY2784544 inhibited 293T-ACE2-TMPRSS2 cell fusion but caused cell aggregation due to cytotoxicity (Figure 8) whereas foretinib displayed a low level of fusion inhibition under high cell density (Supplementary Figure S13). Another serine/threonine kinase inhibitor, cobimetinib (Signorelli and Shah Gandhi, 2017), moderately inhibited fusion luciferase activity under select conditions (Figure 6, Supplementary Figure S12) but which might be partly accounted for by its cytotoxicity (Figure 6). The non-cytotoxic protein kinase C inhibitor, sotrastaurin (Kovarik and Slade, 2010), inhibited fusion luciferase activity in 293T-ACE2-TMPRSS2 cells and pH5-dependent fusion in 293T-ACE2 cells but only showed a low level of fusion inhibition morphologically at high density (Supplementary Figure S13), suggesting that it is a moderate inhibitor. None of the above kinase inhibitors inhibited 293T-ACE2 cell fusion at physiological pH but they (apart from vandetanib) all inhibited 293T-ACE2-TMPRSS2 cell fusion at physiological pH. Overall, kinases are generally moderate inhibitors. Select kinases (MLN8237 and LY2784544) are potent fusion inhibitors but are cytotoxic at the same time.

### CDK and PARP inhibitors are universal fusion inhibitors

We investigated two classes of inhibitors targeting the cyclin-dependent kinases (CDKs) and poly (ADP-ribose) polymerase (PARP). CDK and PARP inhibitors were in common of being universal, potent inhibitors (Figure 6, Supplementary Figure S12). They even inhibited 293T-ACE2 cell fusion at physiological pH, which had been refractory to inhibition by most of the molecules tested, raising the possibility that CDK and PARP inhibitors are non-specific transcriptional inhibitors rather than *bona fide* fusion inhibitors. However, both were not true T7 promoter suppressor (Figure 7). The PARP inhibitors did not suppress the T7 promoter whereas the CDK inhibitors showed some degree of inhibition of the T7 promoter but not the luc/gal ratio. Indeed, most of them showed low levels of fusion inhibition morphologically (Supplementary Figure S13). LY2835219 showed some fusion inhibition by retaining a considerable number of clustered single intact cells amongst small disintegrated syncytia (Figure 8). Dinaciclib inhibited 293T-ACE2-TMPRSS2 cell fusion but its cytotoxicity caused cell aggregation (Figure 8). Due to their universal potency, we studied a non-cytotoxic representative of the CDK and PARP inhibitors, palbociclib HCl and rucaparib, to see whether they mediated this universal inhibition by inhibiting ACE2-spike binding but we did not detect any inhibition of ACE2-spike binding (Figure 9).

### Retinoid does not play a major role in fusion

We previously showed that retinoid exhibited opposing effects on SARS-2-S pseudovirus infection (Chan et al., 2021). In this study, we showed significant but modest inhibition of 293T-ACE2 cell fusion at pH7.4 + trypsin by adapalene and tazarotene and at pH5 by bexarotene (Figure 6, Supplementary Figure S12). Retinoid did not inhibit 293T-ACE2 cell fusion at pH7.4 and pH5+trypsin or 293T-ACE2-TMPRSS2 cell fusion. Overall, retinoid did not seem to play a major role in fusion.

### Oestrogen/progesterone receptor modulators do not inhibit virus fusion

We previously identified several drug hits that are involved in the oestrogen/progesterone pathways (Chan et al., 2021). In this study, we found that the oestrogen receptor modulators, tamoxifen citrate and raloxifene (also a kitelike-shaped molecule, see above) (Yu et al., 2022), did not significantly inhibit fusion luciferase activity under all conditions, suggesting that they are not involved in the fusion step (Figure 6, Supplementary Figure S12). In contrast, the progesterone receptor agonist, drospirenone, specifically enhanced fusion luciferase activity in 293T-ACE2 cells but not in 293T-ACE2-TMPRSS2 cells. Although drospirenone-progesterone receptor complex is a known transcriptional activator (Africander et al., 2011), drospirenone turned out to be a T7 promoter suppressor in our negative screen (Figure 7). Therefore, drospirenone is not a fusion inhibitor but its role as a fusion enhancer remains to be determined.

### Anidulafungin and tenofovir are broad-spectrum and selective fusion inhibitors

We have previously identified some anti-viral, anti-bacterial and anti-fungal with anti-SARS-CoV-2 activity (Chan et al., 2021). Here, we studied whether some of them mediated their anti-viral effects by acting as a fusion inhibitor. The antibiotic, azithromycin, was amongst our top tips in our previous anti-viral screen. Together with simeprevir, a HCV protease inhibitor (Vaidya and Perry, 2013), they showed moderate inhibition of fusion luciferase activity under select conditions (Figure 6, Supplementary Figure S12). Morphologically, they showed a low level of inhibition of 293T-ACE2-TMPRSS2 cell fusion at high cell density (Supplementary Figure S13), suggesting that they are not major fusion inhibitors. Arbidol and anidulafungin inhibited moderate to high levels of fusion luciferase activity under all conditions apart from pH7.4 in 293T-ACE2 cells (Figure 6, Supplementary Figure S12). Arbidol was non-toxic (Figure 6) whereas anidulafungin strongly inhibited both the T7 and CMV promoters in the negative screen despite not being a specific T7 promoter suppressor (Figure 7), suggesting cytotoxicity. Morphologically, Arbidol inhibited a moderate level of fusion in 293T-ACE2-TMPRSS2 (Supplementary Figure S13) whereas anidulafungin inhibited a high level of fusion (Figure 8). The reverse transcriptase (RT) inhibitor, tenofovir disoproxil fumarate (Desai et al., 2017), has been used as a negative control in our previous anti-SARS-CoV-2 screen (Chan et al., 2021). Surprisingly, tenofovir disoproxil fumarate displayed selective potent inhibition of fusion luciferase activity in 293T-ACE2-TMPRSS2 cells and pH7+trypsin fusion in 293T-ACE2 cells (Figure 6, Supplementary Figure S12). Given that it is an adenosine analogue RT inhibitor, it was possible that tenofovir disoproxil fumarate non-specifically inhibited the T7 promoter rather than fusion *per se*. However, tenofovir disoproxil fumarate did not suppress the T7 and CMV promoters in the negative screen (Figure 7) and was non-cytotoxic (Figure 6). Moreover, tenofovir disoproxil fumarate was selective. We confirmed this selectivity in fusion inhibition using morphological criteria by showing fusion inhibition in susceptible 293T-ACE2-TMPRSS2 cells but not in non-susceptible 293T-ACE2 cells under physiological pH (Figure 8). Interestingly, anidulafungin inhibited 53% of ACE2-spike binding whereas tenofovir did not inhibit ACE2-spike binding (Figure 9). Overall, we have identified anidulafungin and tenofovir disoproxil fumarate as broad and selective potent fusion inhibitors, respectively, which might be explained by their differential ability to inhibit ACE2-spike protein binding.

### Natural compounds are not significant fusion inhibitors

Natural compounds are increasingly popular in drug discovery. In this study, we screened four natural compounds previously shown as top hits to inhibit SARS-CoV-2 pseudovirus infection (Chan et al., 2021) for fusion inhibition. These natural compounds all displayed some degree of cytotoxicity (Figure 6) resulting in strong suppression of the T7 and CMV promoters despite not being specific T7 promoter suppressors (Figure 7). This cytotoxicity might account for some of the reduction in fusion luciferase activity, as illustrated by tetrandrine (see above), gossypol and timosaponin A3 (Figure 6, Supplementary Figure S12). Morphologically, gossypol displayed a low level of fusion inhibition in 293T-ACE2-TMPRSS2 cells (Supplementary Figure S13). Timosaponin A3 was very cytotoxic resulting in rounding off and detachment of cells. Therefore, its fusion inhibition activity could not be assessed. In contrast, oridonin did not inhibit but rather increased fusion in some cases. Overall, the natural compounds studied are not significant fusion inhibitors.

### Select fusion inhibitors inhibit infection of TMPRSS2(+) cells

**FIGURE 10:**
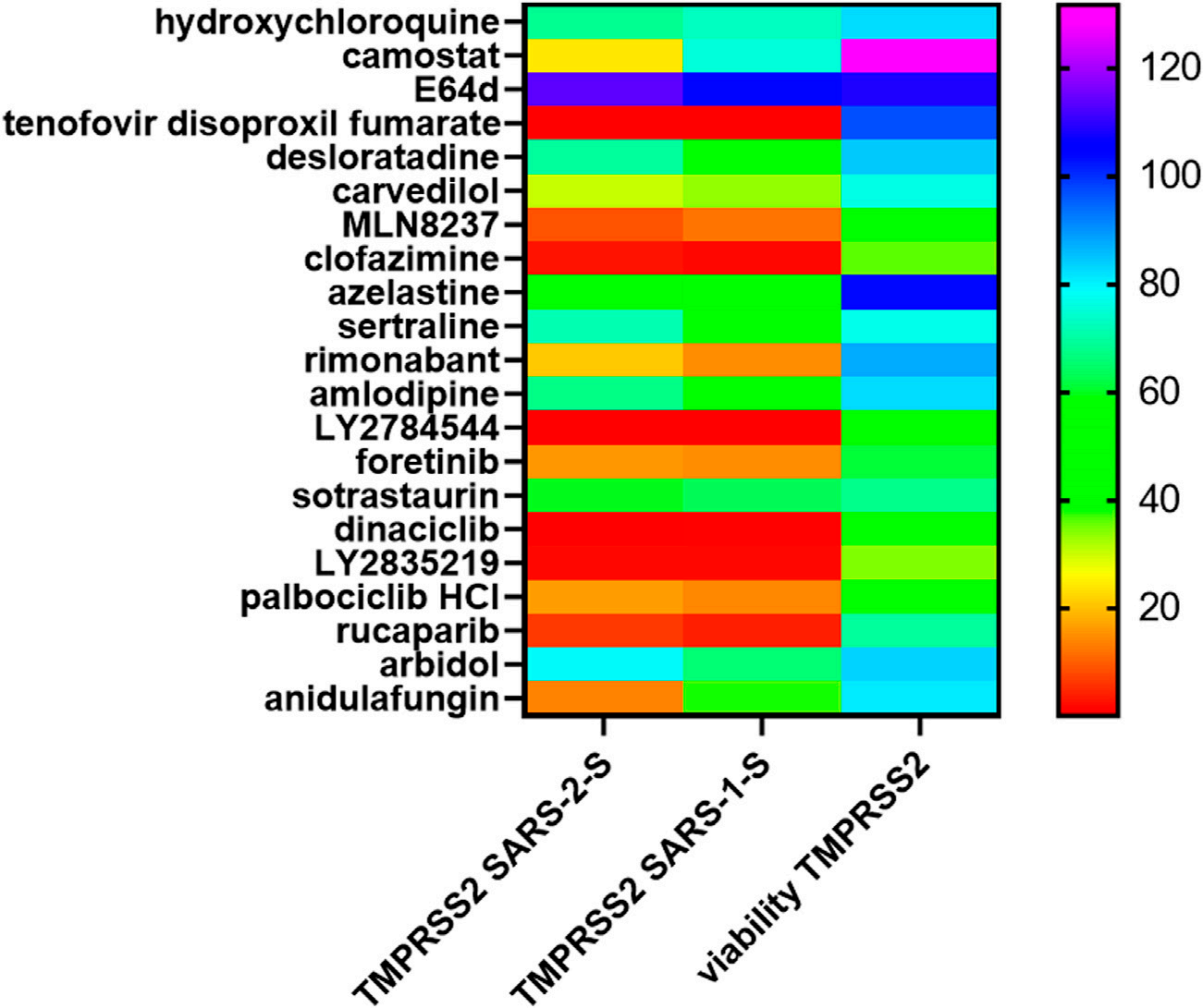
Rimonabant, rucaparib, carvedilol and anidulafungin inhibit SARS-CoV-2 infection of TMPRSS2 cells. Mouse leukaemia virus pseudotyped with the spike protein (S) from severe acute respiratory syndrome coronavirus (SARS-1-S) and SARS-CoV-2 (SARS-2-S) was used to infect 293T-ACE2-TMPRSS2 cells, in a 96-well plate for 48 h in the presence of the drug, as indicated, with 1 h pre-treatment. Infectivity was measured as luciferase activity and expressed as % infectivity *versus* infected, solvent control. Viability was measured by XTT assays in un-infected samples and expressed as % viability *versus* solvent control. Data are presented as a heat map of the mean of three repeats.

These drug hits were discovered in our previous study using pseudovirus infection of 293T-ACE2, therefore, they are all known inhibitors of the endosomal entry pathway (Chan et al., 2021). In this study, many of these drug hits inhibited 293T-ACE2-TMPRSS2 cell fusion at physiological pH, therefore, we sought to confirm whether they also inhibited the plasma membrane entry pathway. SARS-2-S pseudovirus infection of 293T-ACE2-TMPRSS2 cells was potently inhibited by the TMPRSS2 inhibitor, camostat, but not by the cathepsin L inhibitor, E64d, or the lysosomotropic agent, hydroxychloroquine, confirming that SARS-CoV-2 infection of TMPRSS2(+) cells is mediated *via* the plasma membrane fusion pathway (Figure 10). After excluding cytotoxic drug hits from our analysis, only rucaparib and rimonabant showed potent inhibition of both SARS-2-S and SARS-1-S pseudovirus infection of 293T-ACE2-TMPRSS2 cells. The lopsided kite, carvedilol, inhibited ∼70% of SARS-2-S and SARS-1-S pseudovirus infections. Anidulafungin showed potent inhibition of SARS-2-S and 70% inhibition of SARS-1-S infections.

The ability to inhibit infections of both the endosomal (Chan et al., 2021) and plasma membrane routes (above) may involve targeting a common step e.g. ACE2-spike protein binding. Indeed, rimonabant and anidulafungin inhibited ACE2-spike protein binding (Figure 9). In contrast, carvedilol and rucaparib did not inhibit ACE2-spike protein binding, suggesting that they target a different step common to both endosomal and plasma membrane entry.

### Select fusion inhibitors exhibit pan-coronaviral or pan-viral inhibition

**FIGURE 11:**
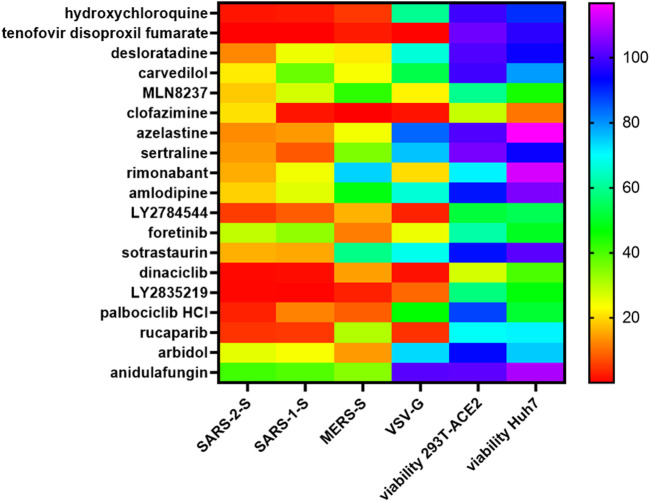
Pan-coronaviral and pan-viral fusion inhibitors. Mouse leukaemia virus pseudotyped with glycoprotein from vesicular stomatitis virus (VSV-G) and spike protein (S) from severe acute respiratory syndrome coronavirus (SARS-1-S), SARS-CoV-2 (SARS-2-S) and Middle East respiratory syndrome coronavirus (MERS-S), was used to infect 293T-ACE2 (for SARS-1-S, SARS-2-S, VSV-G) or Huh-7 (for MERS-S) cells, in a 96-well plate for 48 h in the presence of the drug, as indicated, with 1 h pre-treatment. Infectivity was measured as luciferase activity and expressed as % infectivity *versus* infected, solvent control. Viability was measured by XTT assays in un-infected samples and expressed as % viability *versus* solvent control. Data are presented as a heat map of the mean of three repeats.

To test the pan-coronaviral and pan-viral potential of these fusion inhibitors, we examined their inhibitory effects against SARS-1-S, Middle East respiratory syndrome coronavirus spike protein (MERS-S) and VSV-G pseudoviruses. The lysosomotropic agent, hydroxychloroquine, potently inhibited SARS-2-S, SARS-1-S and MERS-S pseudovirus infections which fuse at late endosome/lysosome but only modestly inhibited VSV-G infection which fuses at early endosome, confirming the specificity of this assay (Figure 11). After excluding cytotoxic drug hits from our analysis, we found that carvedilol, anidulafungin together with desloratadine, azelastine and arbidol were pan-coronavirus with azelastine and desloratadine the most potent. Sotrastaurin, sertraline and amlodipine were SARS-CoVs-specific whereas sertraline and amlodipine also exhibited moderate/modest inhibition of MERS-S pseudovirus infection. Rimonabant and rucaparib were peculiar in exhibiting potent cross-family inhibition of SARS-2-S, SARS-1-S and VSV-G pseudovirus infections but only moderate/no inhibition of MERS-S pseudovirus infection. The ability of rimonabant to inhibit VSV-G indicates that it also acts on other step in addition to ACE2-spike binding.

## Discussion

Fusion is structurally and mechanistically conserved and is a prime target for pan-viral therapeutics (Vigant et al., 2015). Fusion can be broadly divided into plasma membrane and endosomal (Eckert and Kim, 2001). Virus family and individual viruses have evolved to use either one of the pathways but some viruses have the flexibility to switch from one to the other (Belouzard et al., 2012; Ou et al., 2021). At the beginning of the COVID-19 pandemic, it was believed that the SARS-CoV-2 utilizes the plasma membrane fusion as the default pathway but can switch to the endosomal pathway in the absence of the plasma membrane protease TMPRSS2 (Hoffmann et al., 2020). The omicron variant preferentially uses the endosomal pathway even in the presence of the TMPRSS2 (Meng et al., 2022), suggesting evolution towards endosomal pathway. Syncytia formation has emerged to be a significant pathological features in COVID-19 patients and may account for SARS-CoV-2 pathogenesis, virus spread and immune evasion (Leroy et al., 2020; Braga et al., 2021). The alpha, beta and delta variants are more fusogenic than the omicron variant (Arora et al., 2021; Rajah et al., 2021; Zhang et al., 2021; Meng et al., 2022; Suzuki et al., 2022). It is, therefore, important to find an anti-viral which can broadly inhibit different modes of fusion. This requires assays that have the capacity for sensitive and rapid screening and adaptability. Here, we have developed fusion assays that can distinguish between TMPRSS2-dependent plasma membrane, TMPRSS2-independent alternative fusion and syncytia formation. In addition, we have identified putative, acidic pH-dependent and pH-, trypsin-dependent fusion mechanisms. Screening against our top drug hits from our previous anti-SARS-CoV-2 infection screen (Chan et al., 2021) illustrated the sensitivity of our reporter gene assay over that of morphological fusion assay. Whilst our reporter gene assay can distinguish between potent, moderate and modest fusion inhibitors, the morphological assay can only detect potent fusion inhibition corresponding to reporter gene activity of <0.5. Therefore, we have established sensitive, rapid and adaptable reporter gene assays to screen for inhibitors of fusion and syncytia formation induced by various triggers. The use of transient transfection normally results in a higher expression of the spike protein and hence more fusion which will make this fusion assay more stringent in the detection of potent inhibitors. It has the flexibility of converting into a stable cell system to offer simplicity, speed and standardization.

Interestingly, all modes of fusion are potently inhibited by hydroxychloroquine, chloroquine and batimastat. Hydroxychloroquine/chloroquine alkylation of endosome/lysosome to inhibit fusion/autophagy (Gies et al., 2020) is not applicable as we are detecting cell-cell surface fusion. We showed that hydroxychloroquine did not inhibit ACE2-spike binding (Figure 9). It remains to be determined whether hydroxychloroquine and chloroquine interfere with ACE2 glycosylation or other unknown mechanisms (Gies et al., 2020; Wang et al., 2020). The MMPs are emerging to play a subsidiary role to the TMPRSS2- and endosomal-mediated entry (Nguyen et al., 2020; Jocher et al., 2022; Yamamoto et al., 2022). ADAM10 was found to be responsible for fusion in A549-ACE2 cells and in HEC50B-TMPRSS2 cells (Jocher et al., 2022; Yamamoto et al., 2022). It is, therefore, not surprising to find that fusion in 293T-ACE2 cells and 293T-ACE2-TMPRSS2 cells were inhibited by batimastat in this study. This is somewhat contradictory to the findings of Hornich and co-workers who found that batimastat inhibited syncytia formation at physiological pH in 293T-ACE2 cells but not in 293T-ACE2-TMPRSS2 cells (Hörnich et al., 2021). This discrepancy may be explained by the use of transient transfection of ACE2 and TMPRSS2 in their study and the use of stable cell lines in our study. Our results are supported by the demonstration of co-existence of an MMP, TMPRSS2 infection pathway in HEC50B-TMPRSS2 cells (Yamamoto et al., 2022).

Whilst an acidic pH induces conformational changes of the envelope protein in other viruses to facilitate endosomal fusion (White and Whittaker, 2016), SARS-CoVs are unique in that they utilize an acidic pH to activate an endosomal protease-cathepsin L (Simmons et al., 2005; Ou et al., 2020). Therefore, an acidic pH is not strictly required for fusion to occur as long as the protease has been pre-activated. In this study, we showed that pH5-induced fusion was distinctive from that of fusion at physiological pH in their inhibition profiles. It could be explained by acidic pH-induced conformational changes as in other viruses (White and Whittaker, 2016) or by acidic pH-activated cathepsin L or other proteases. Emerging evidence suggests that SARS-CoV-2 requires an acidic pH for plasma membrane fusion (Kreutzberger et al., 2022) and (together with Ca^2+^) to cause conformation changes in fusion (Singh et al., 2022). Although cathepsin L is an endosomal protease, it can also be secreted and activated in an acidic environment (Fonović and Turk, 2014). SARS-CoV-2 infection has been found to increase expression of cathepsin L in liver cells *in vitro* and *in vivo* (Zhao et al., 2021). Increased levels of circulating cathepsin L were detected in patients with severe COVID-19 than in patients with non-severe COVID-19 or in healthy individuals. It is, therefore, not unlikely that this kind of fusion occurs *in vivo*, given the right environment. Other candidate includes pH-activated exogenous proteases. We noticed that pH5-induced fusion was more susceptible to batimastat inhibition than that induced by other conditions. It is, therefore, tempting to speculate the involvement of an acidic pH-activated MMP (Davis, 1991; Gunja-Smith and Woessner, 1993).

The discovery of non-canonical cleavage sites and proteases points to the likelihood of alternative fusion mechanisms and our detection of pH 5 + trypsin-triggered fusion may be one of them. Furin cleaves at the canonical S1/S2 site and also at and near the S2′ site (Essalmani et al., 2022; Zhang et al., 2022). TMPRSS2 cleaves at multiple sites including the S1/S2 and S2’ (Essalmani et al., 2022; Fraser et al., 2022). Cathepsin L cleaves at T678 downstream of the S1/S2 site in SARS-1-S (Bosch et al., 2008) and at the N-terminal domain and upstream of the S1/S2 in SARS-2-S (Zhao et al., 2022). Trypsin cleaves at multiple sites within the S1/S2 (Jaimes et al., 2020; Mustafa et al., 2021) and likely the S2′ R797 of SARS-2-S in analogous to the SARS-1-S (Belouzard et al., 2009). ADAM10 cleaves the SARS-2-S at or near the S2’ site and near the S1/S2 site (Hörnich et al., 2021; Jocher et al., 2022; Yamamoto et al., 2022). Neutrophil elastase and protease 3 cleave right downstream of the polybasic site (Mustafa et al., 2021).

The fusion inhibitors we identified tend to scatter across pharmacological groups rather than concentrate in particular pharmacological groups. Nevertheless, we have excluded several pharmacological groups (retinoid, oestrogen/progesterone modulators) in the fusion process. Using pseudovirus infection, we have previously identified a group of kite-shaped and kitelike-shaped molecules as our top hits (Chan et al., 2021). Kite-shaped molecules are a class of molecules that displayed a similar structure and a shape reminiscent of a traditional Chinese kite. These had a well-conserved tri-cyclic core structure (forming the sail of the kite) and a more variable extension from the central 6- or 7-membered ring (forming the tail of the kite). They are mostly anti-psychotics, anti-depressants and anti-histamines that target the serotonin 5-hydroxytryptamine, dopamine, H1 histamine, muscarinic and adrenergic receptors. In this study, we found that selective kite-shaped and kitelike-shaped molecules are moderate fusion inhibitors under select conditions. On the other hand, lopsided kites are potent inhibitors. Interestingly, they belong to three pharmacologically diverse drug classes, suggesting that they share common fusion inhibitory structural motifs on a kite-shaped backbone. Although the lopsided kite, carvedilol, is known to decrease ACE2 expression in endothelial cells (Skayem and Ayoub, 2020); its selective fusion inhibitory activity hints at a structural rather than functional mechanism. Significantly, our identification of the lopsided kite, clofazimine, as a potent fusion inhibitor demonstrates the power of our fusion assays in identifying broad-spectrum anti-virals; as exemplified by its ability to inhibit pan-coronavirus infections in another study (Yuan et al., 2021). Clofazimine has been found to reduce calcium oscillations and phosphatidylserine externalization (Braga et al., 2021), making it a good candidate as a pan-viral drug. By screening a small number of molecules we have already identified structural relatedness in some of the fusion inhibitors. This information is encouraging for the future screening of a larger library to recognize and characterize the common structural features of fusion inhibitors which will eventually lead to the creation of a very efficient pharmacophore.

The nucleoside analogue, tenofovir disoproxil fumarate is an HIV drug (Desai et al., 2017). It has entered into COVID-19 clinical trials but so far has yet to prove its efficacy both clinically (Parienti et al., 2021; Nomah et al., 2022), in small animal models (Park et al., 2020), *ex vivo* and *in vitro* (Choy et al., 2020; Feng et al., 2022). Our identification of tenofovir disoproxil fumarate as a cell type-specific potent fusion inhibitor may open a new avenue for development of tenofovir disoproxil fumarate in synergistic therapeutics.

Inhibition of fusion can involve multiple steps from ACE2-spike binding to fusion *per se*. Broad-spectrum anti-virals are expected to derive from *bona fide* fusion inhibitors that target conserved fusion sites/processes and even physicochemical features of fusion (Vigant et al., 2015). We found that anidulafungin is a potent fusion inhibitor under most conditions; which might be explained by its ability to inhibit ACE2-spike binding. *In silico* analysis also predicted that anidulafungin inhibits ACE2-spike binding by complexing with the less conserved RBD (Ahamad et al., 2022). Therefore, further studies are required to delineate the mechanisms of inhibition before employing our fusion hits into broad-spectrum anti-virals. On the other hand, these small molecules may provide invaluable tools to study the fusion process by trapping intermediates.

Interestingly, our fusion assays also discovered putative fusion enhancer, drospirenone and oridonin. Further work is required to determine whether drospirenone and oridonin enhance fusion non-specifically or only in the presence of SARS-2-S. In the latter case, it is tempting to speculate that they may increase ACE2 expression or bind to the spike protein to facilitate conformational changes. Future work is required to understand the mechanisms of fusion enhancement.

In conclusion, we have developed SARS-2-S, ACE2-specific fusion assays for rapid screening of fusion inhibitors in TMPRSS2(+) and TMPRSS2(-) cell types under different pH and protease activation conditions. The fusion assays are adaptable to enable studying the triggers of the fusion process, which has emerged to be more diverse in SARS-CoV-2 than originally thought. Coupled with the fusion inhibition profiles generated from our top hits of known entry inhibitors, we have identified several new, putative fusion triggers. Our results from the current study suggest that some of our top hits from our previous infection screen (Chan et al., 2021) are inhibiting the fusion process whereas others e.g. kite-shaped molecules are not. Amongst the fusion inhibitors, only a subset inhibits ACE2-spike binding. This will guide us to future studies to identify other mechanisms of entry/fusion inhibition e.g. spike binding, protease inhibition, cell signaling. In perspective, the end results are to create a catalogue of inhibitors with defined targets to be used in synergistic inhibition.

## Data Availability

The original contribution presented in the study are included in the article/Supplementary Material, further inquiries can be directed to the corresponding author.
