# Supplementary figures and images for: Fusion assays for screening of fusion inhibitors targeting SARS-CoV-2 entry and syncytia formation
Source: Front Pharmacol. 2022 Nov 11;13:1007527. doi: 10.3389/fphar.2022.1007527 (PMC9691968; doi:10.3389/fphar.2022.1007527)

## Slide 1
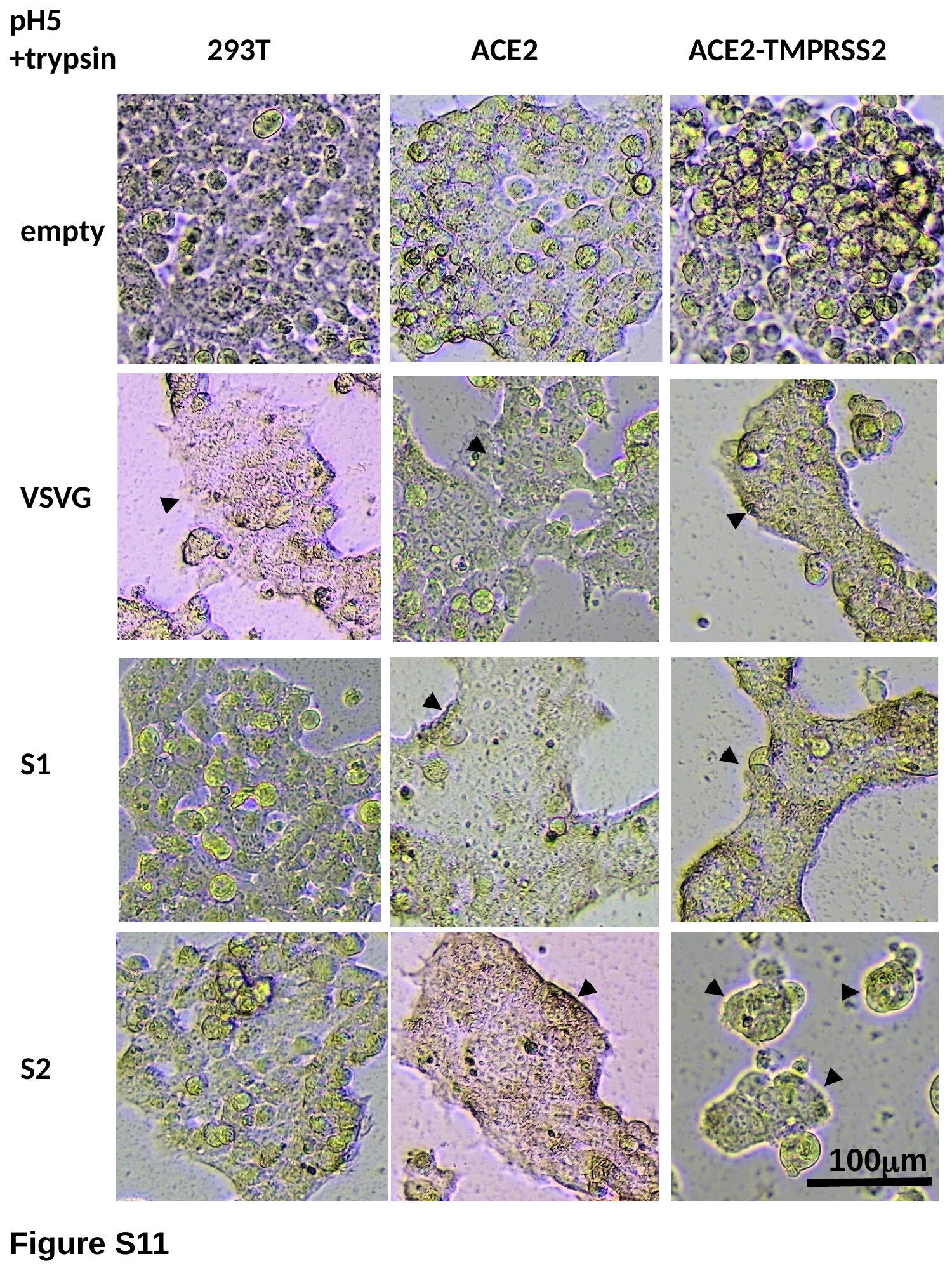

pH5
+trypsin
293T ACE2 ACE2-TMPRSS2
empty
VSVG
S1
S2
100mm
Figure S11

Supplement: Supplementary file 1 [file Presentation9.pptx]

## Slide 1
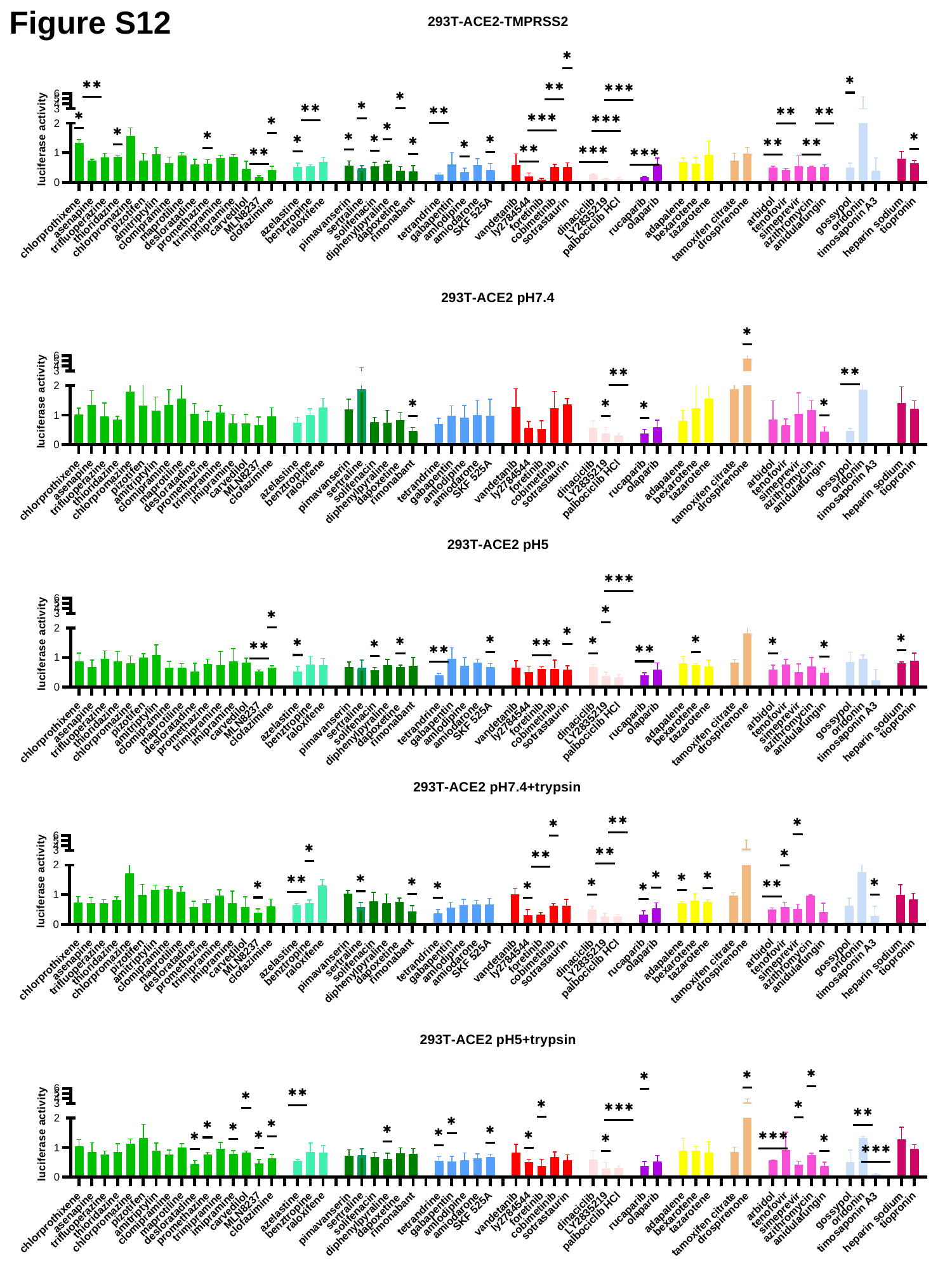

Figure S12

Supplement: Supplementary file 2 [file Presentation10.pptx]

## Slide 1
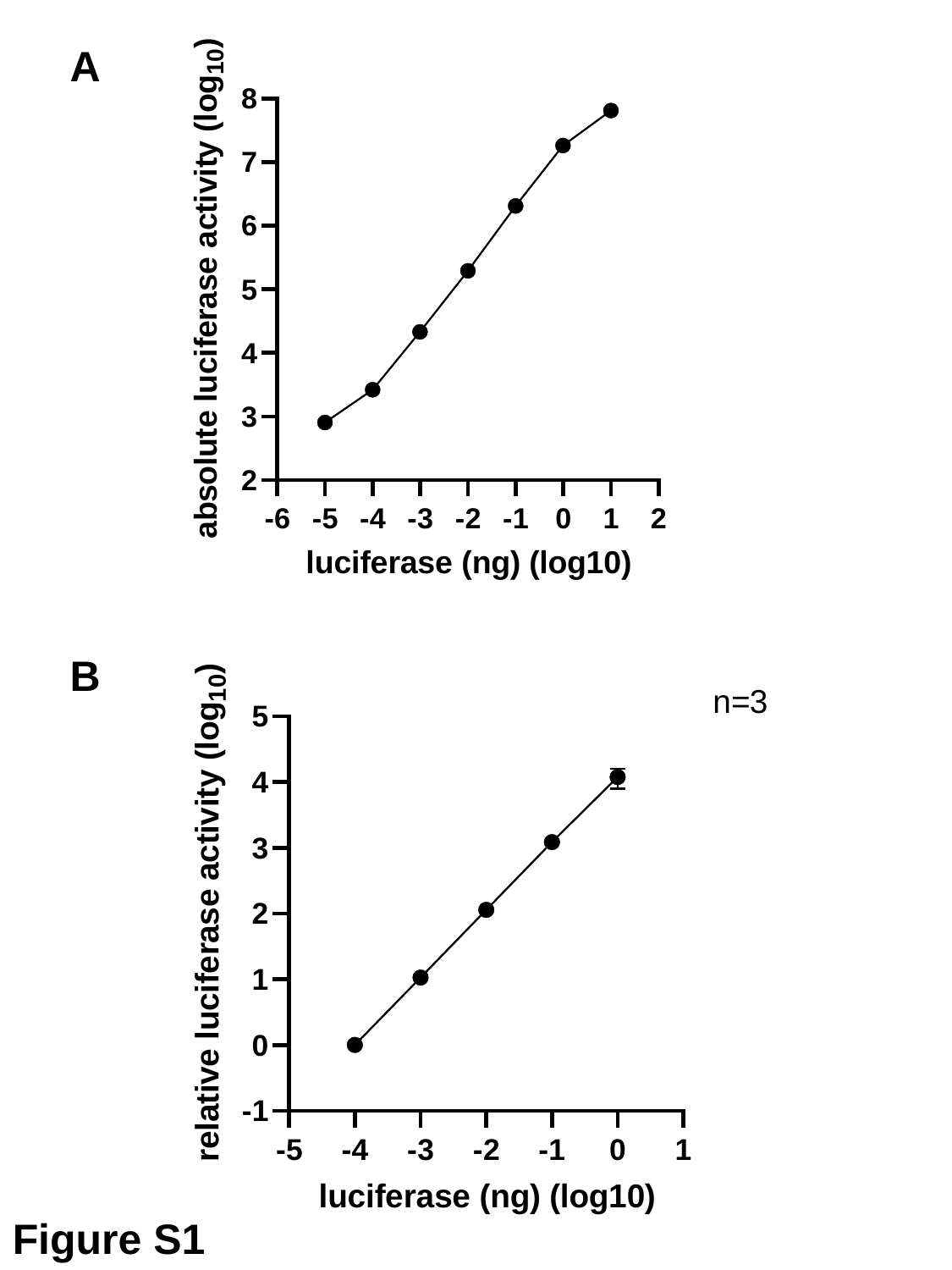

A
B
Figure S1

Supplement: Supplementary file 3 [file Presentation1.pptx]

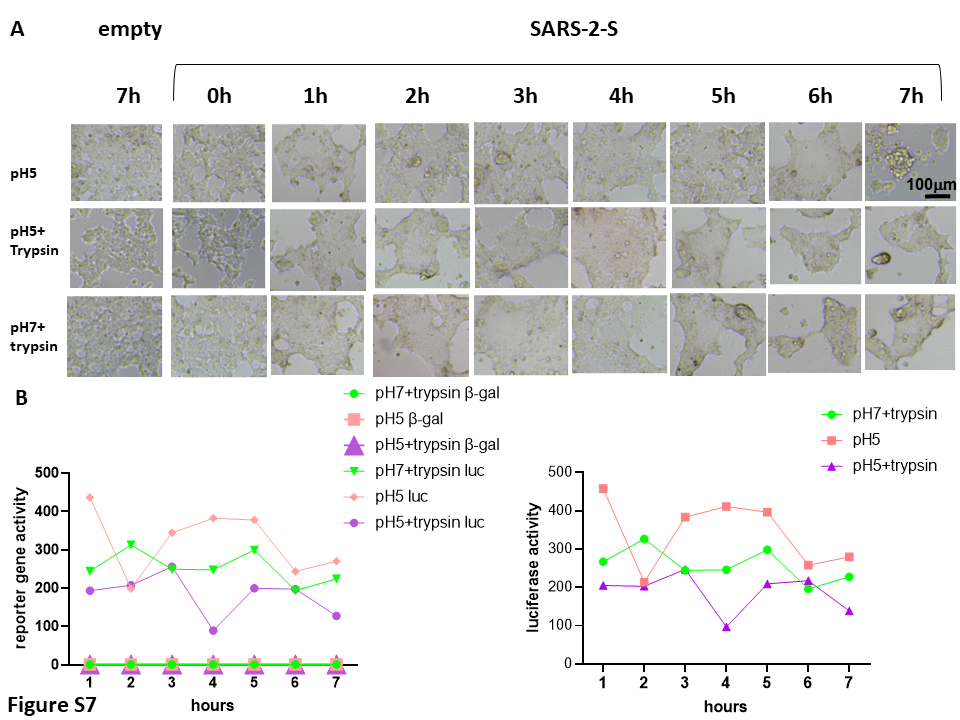

Supplement: Supplementary file 4 [file Image2.tif]

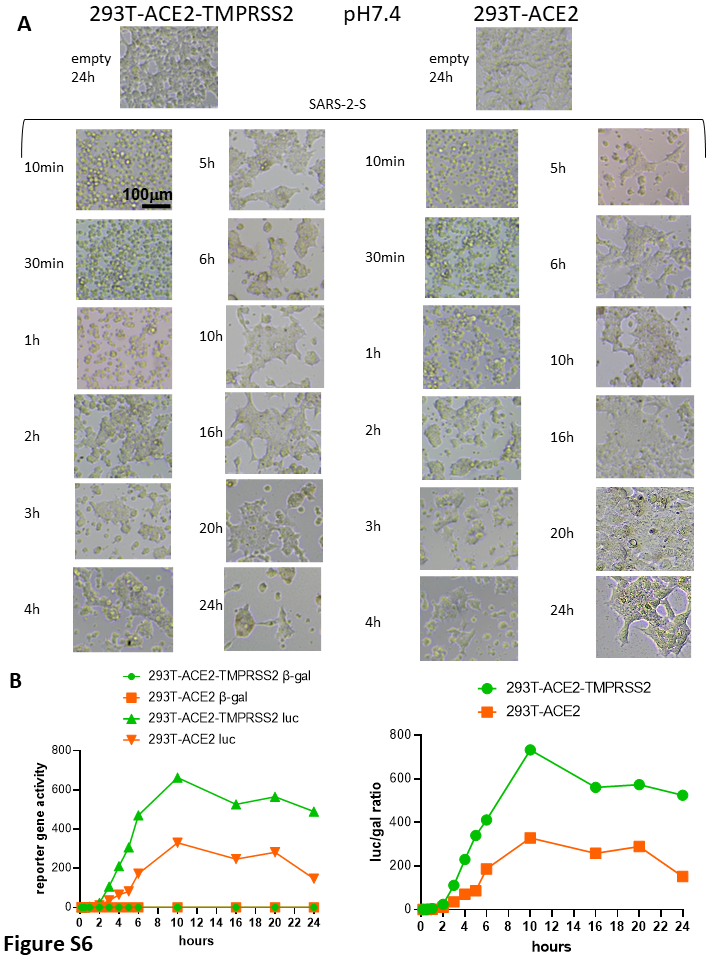

Supplement: Supplementary file 5 [file Image1.tif]

## Slide 1
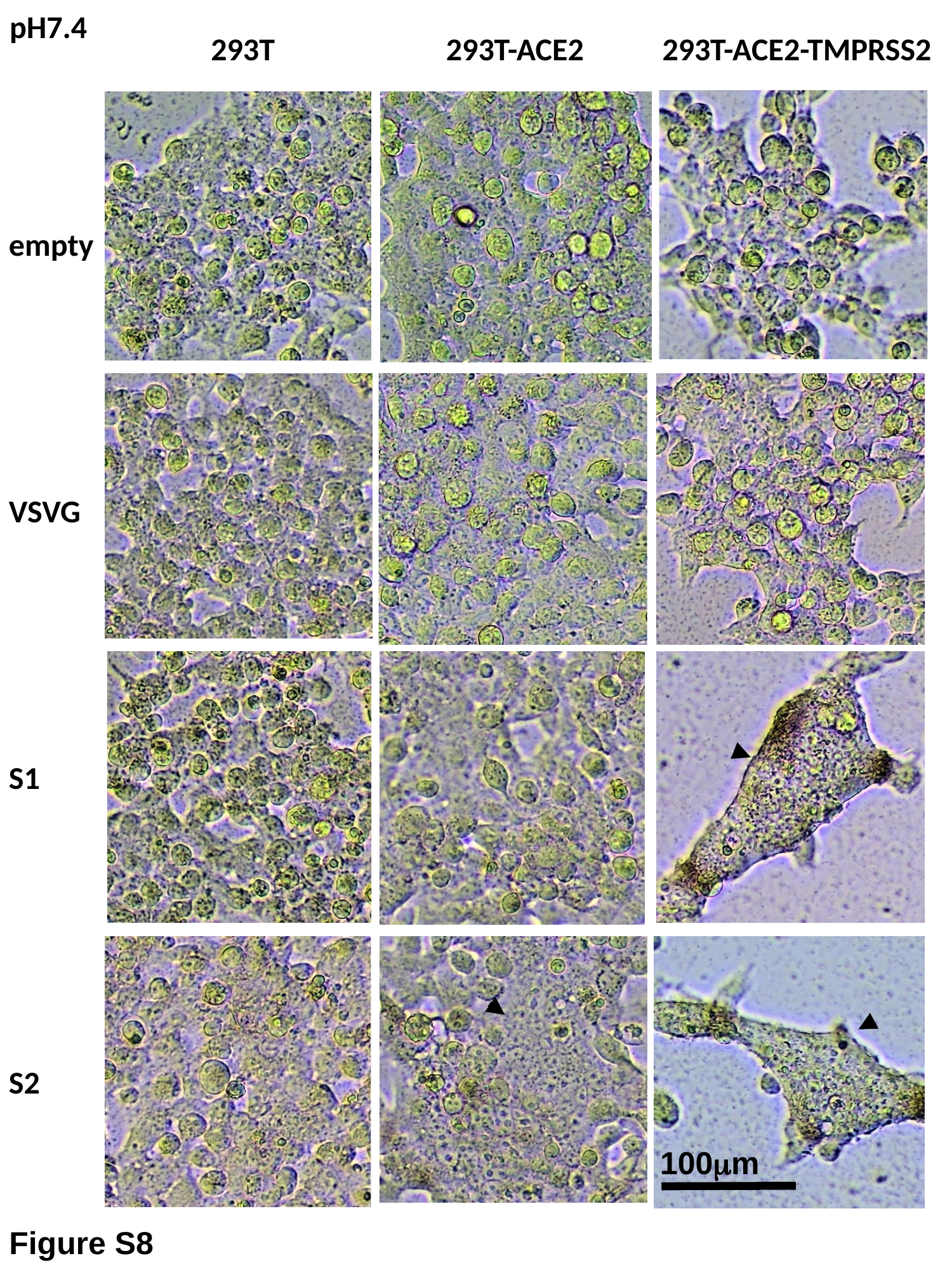

pH7.4
293T 293T-ACE2 293T-ACE2-TMPRSS2
empty
VSVG
S1
S2
100mm
Figure S8

Supplement: Supplementary file 7 [file Presentation6.pptx]

## Slide 1
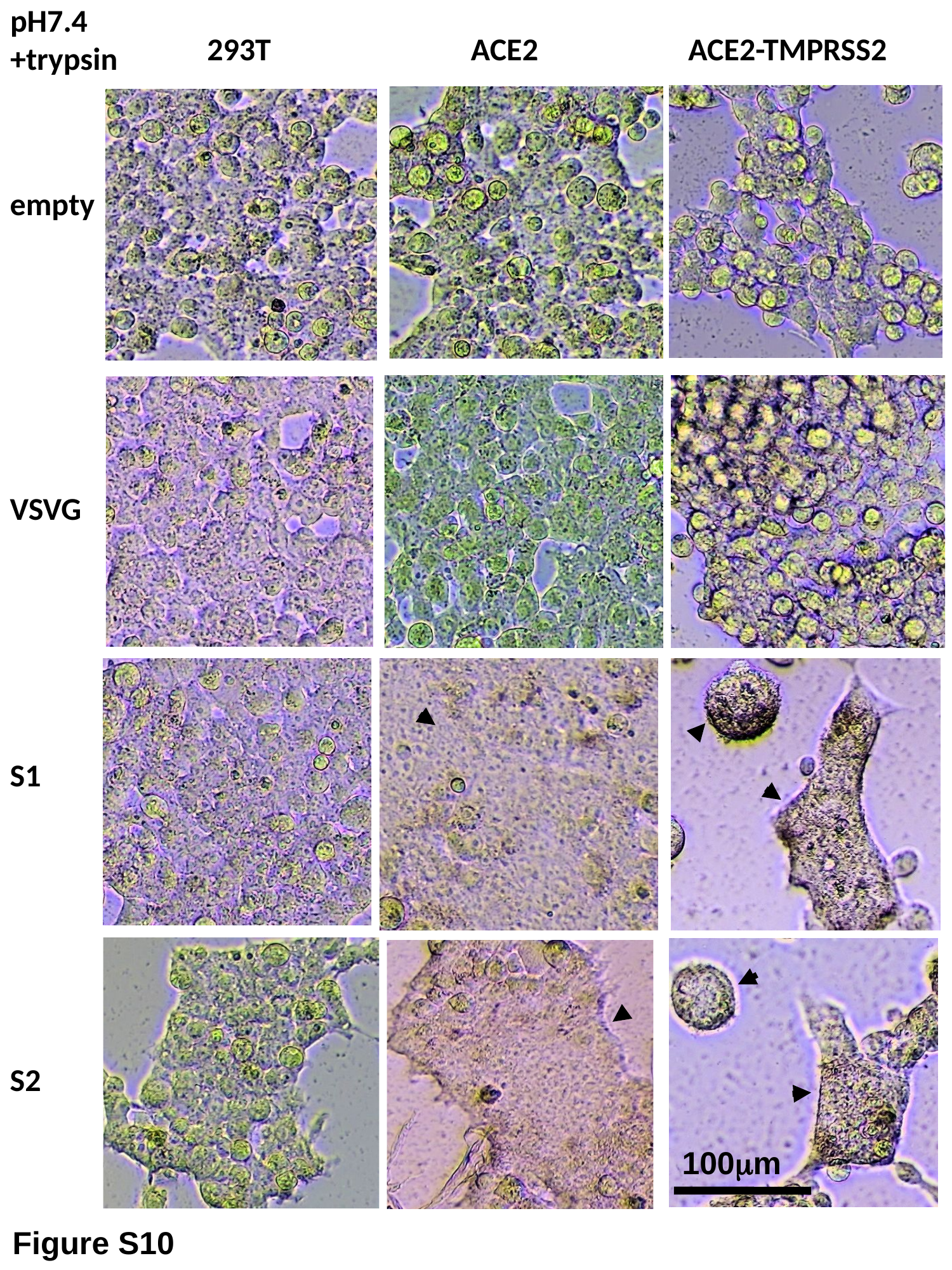

pH7.4
+trypsin
293T ACE2 ACE2-TMPRSS2
empty
VSVG
S1
S2
100mm
Figure S10

Supplement: Supplementary file 8 [file Presentation8.pptx]

## Slide 1
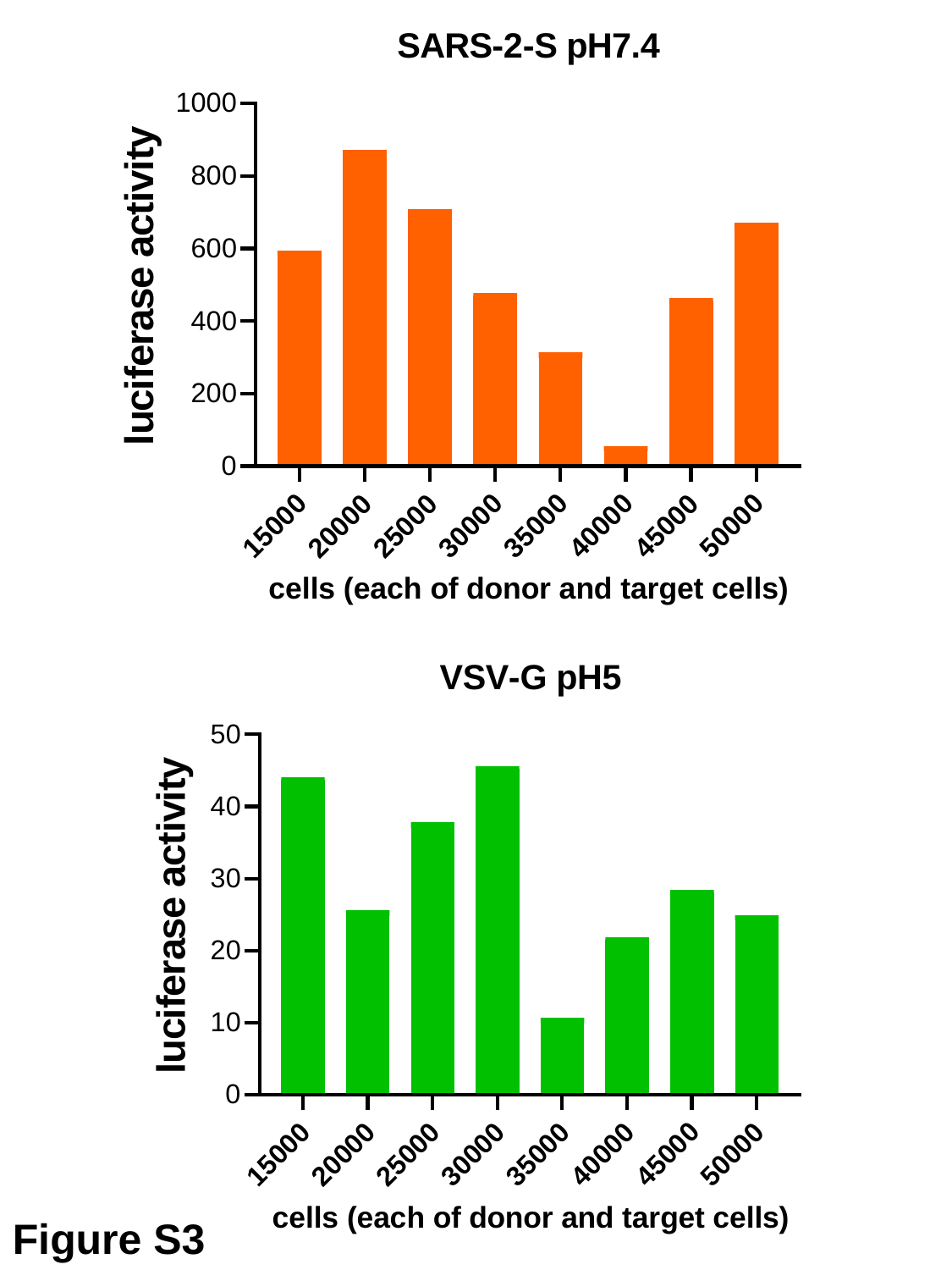

Figure S3

Supplement: Supplementary file 9 [file Presentation3.pptx]

## Slide 1
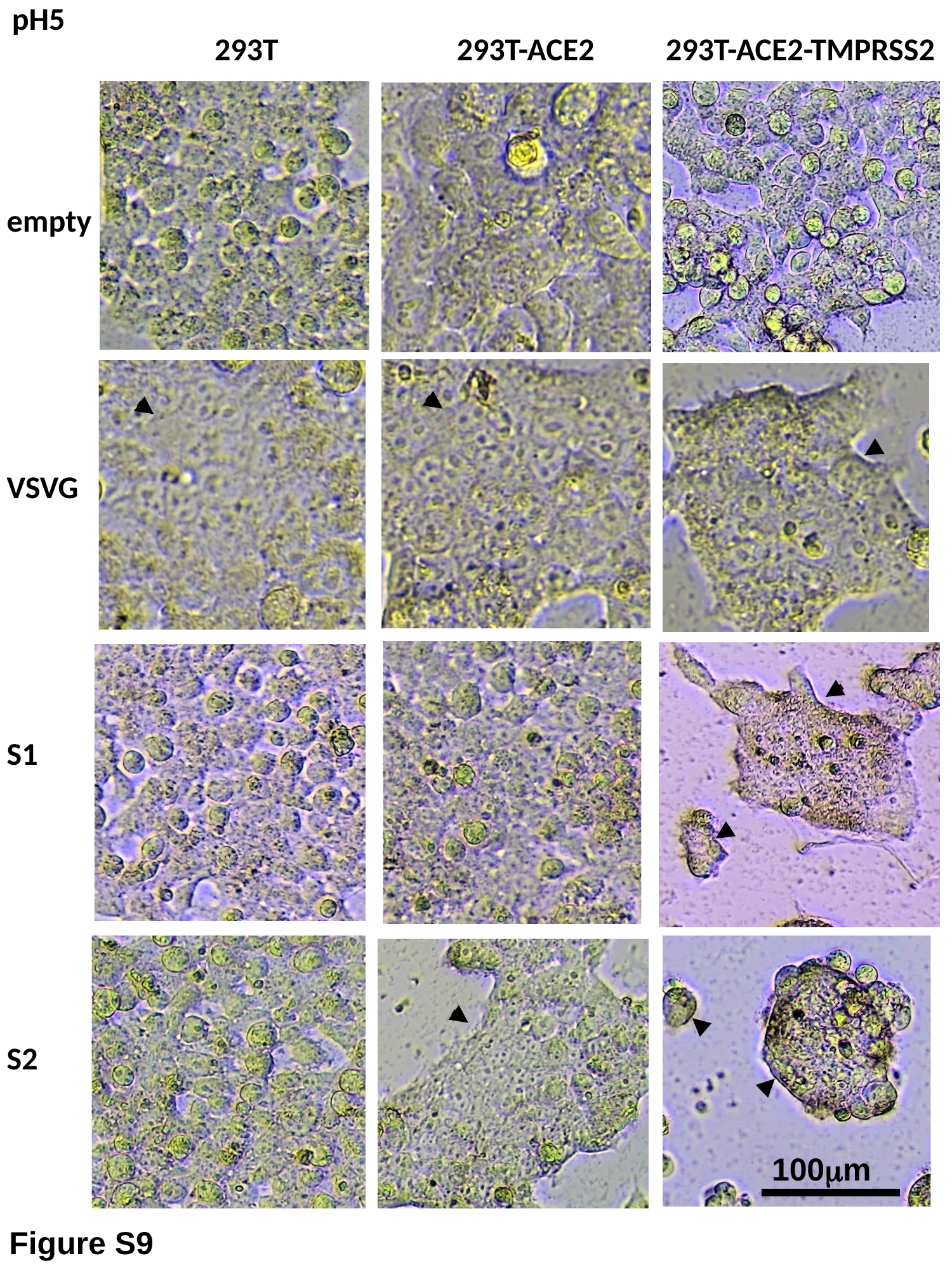

pH5
293T 293T-ACE2 293T-ACE2-TMPRSS2
empty
VSVG
S1
S2
100mm
Figure S9

Supplement: Supplementary file 11 [file Presentation7.pptx]

## Slide 1
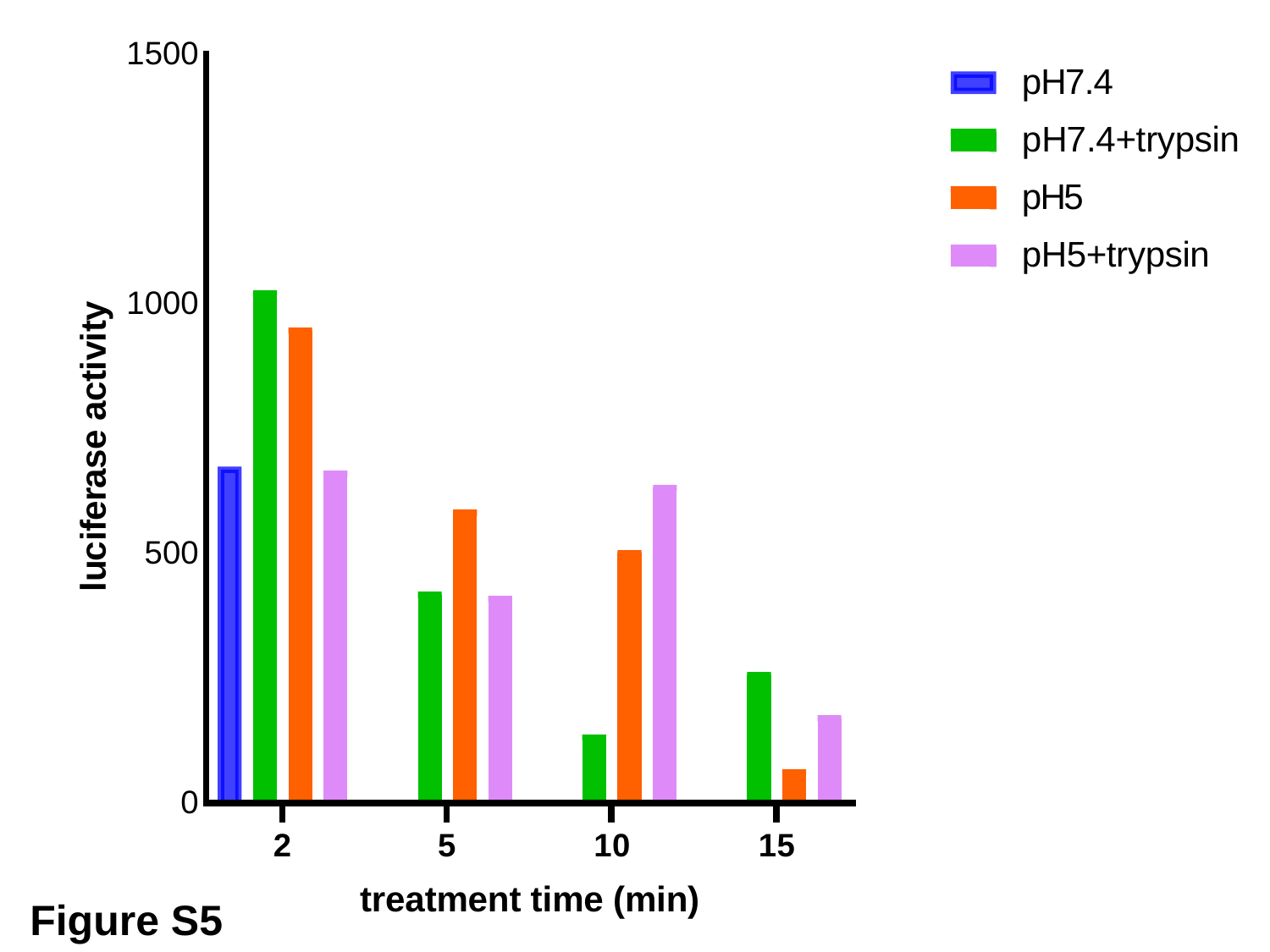

Figure S5

Supplement: Supplementary file 14 [file Presentation5.pptx]
