# Supplementary material for: Fusion assays for screening of fusion inhibitors targeting SARS-CoV-2 entry and syncytia formation
Source: Front Pharmacol. 2022 Nov 11;13:1007527. doi: 10.3389/fphar.2022.1007527 (PMC9691968; doi:10.3389/fphar.2022.1007527)
Supplement: Supplementary file 6 [file Presentation4.pptx]

## Slide 1
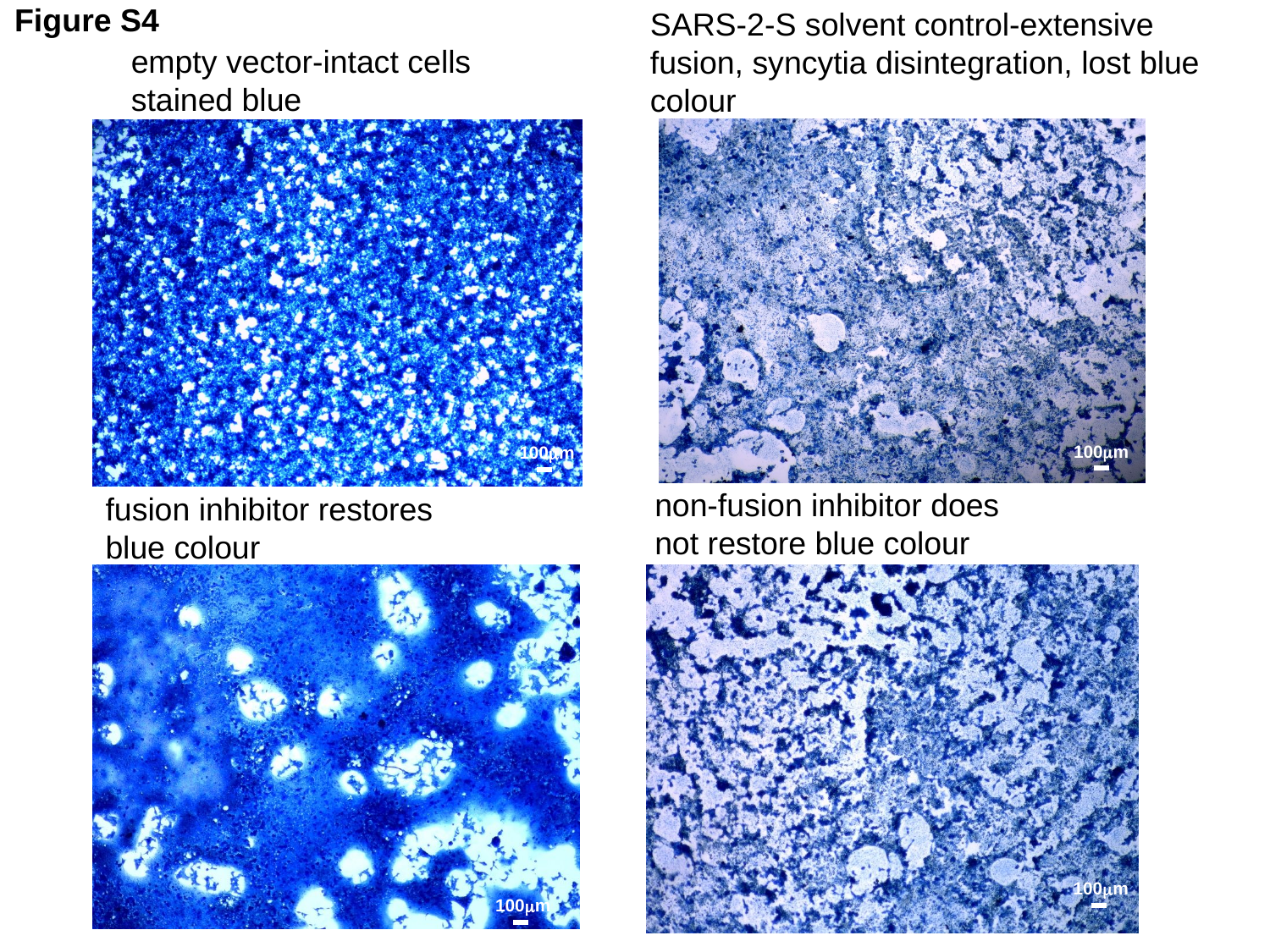

Figure S4
SARS-2-S solvent control-extensive fusion, syncytia disintegration, lost blue colour
empty vector-intact cells
stained blue
100mm
100mm
non-fusion inhibitor does not restore blue colour
fusion inhibitor restores blue colour
100mm
100mm
