# Supplementary material for: Fusion assays for screening of fusion inhibitors targeting SARS-CoV-2 entry and syncytia formation
Source: Front Pharmacol. 2022 Nov 11;13:1007527. doi: 10.3389/fphar.2022.1007527 (PMC9691968; doi:10.3389/fphar.2022.1007527)
Supplement: Supplementary file 10 [file Presentation2.pptx]

## Slide 1
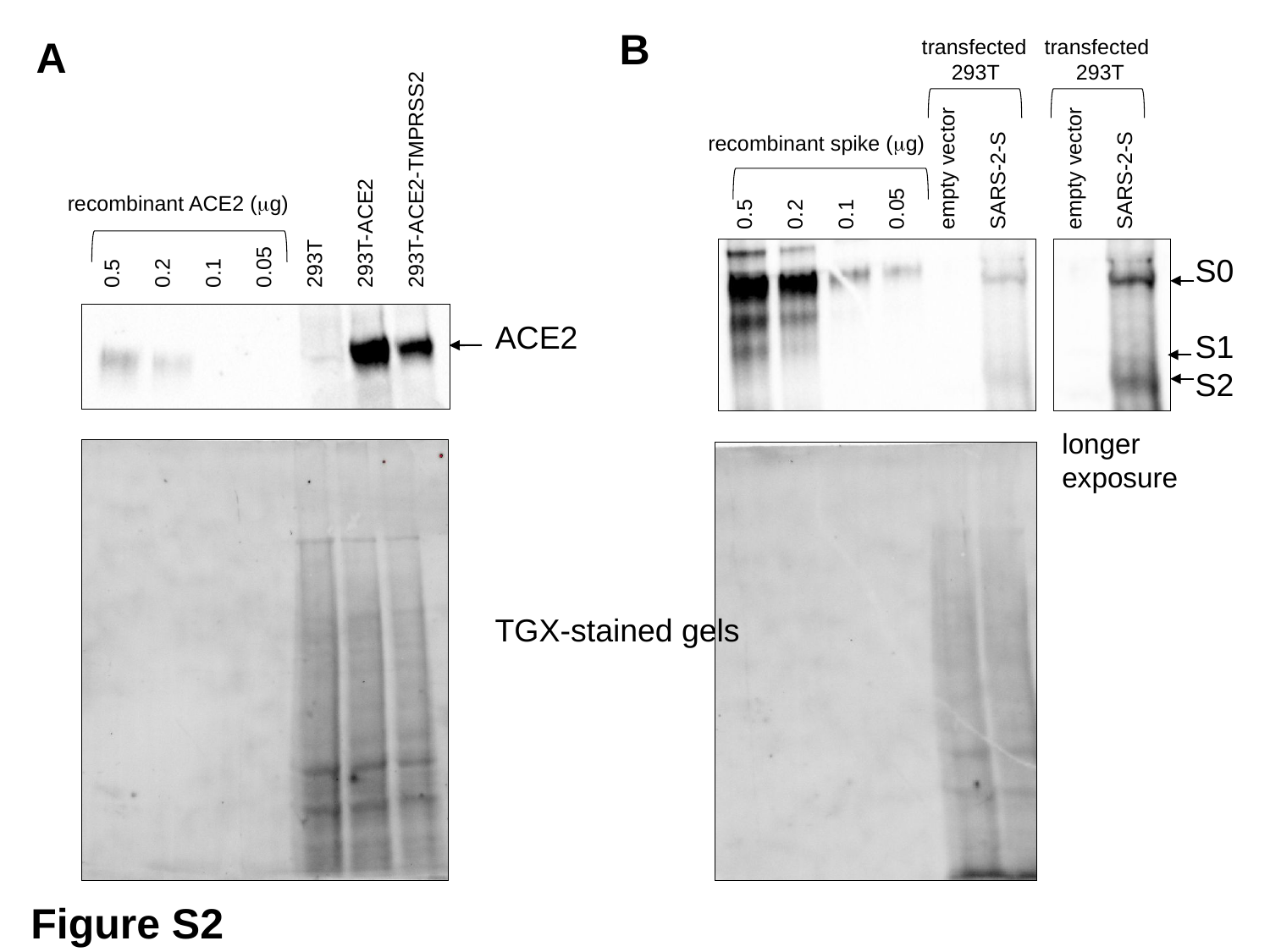

0.5
0.2
0.1
0.05
empty vector
SARS-2-S
empty vector
SARS-2-S
0.5
0.2
0.1
0.05
293T
293T-ACE2
293T-ACE2-TMPRSS2
B
A
transfected transfected
 293T 293T
recombinant spike (mg)
recombinant ACE2 (mg)
S0
S1
S2
ACE2
longer
exposure
TGX-stained gels
Figure S2
