# Supplementary material for: Fusion assays for screening of fusion inhibitors targeting SARS-CoV-2 entry and syncytia formation
Source: Front Pharmacol. 2022 Nov 11;13:1007527. doi: 10.3389/fphar.2022.1007527 (PMC9691968; doi:10.3389/fphar.2022.1007527)
Supplement: Supplementary file 12 [file Presentation11.pptx]

## Slide 1
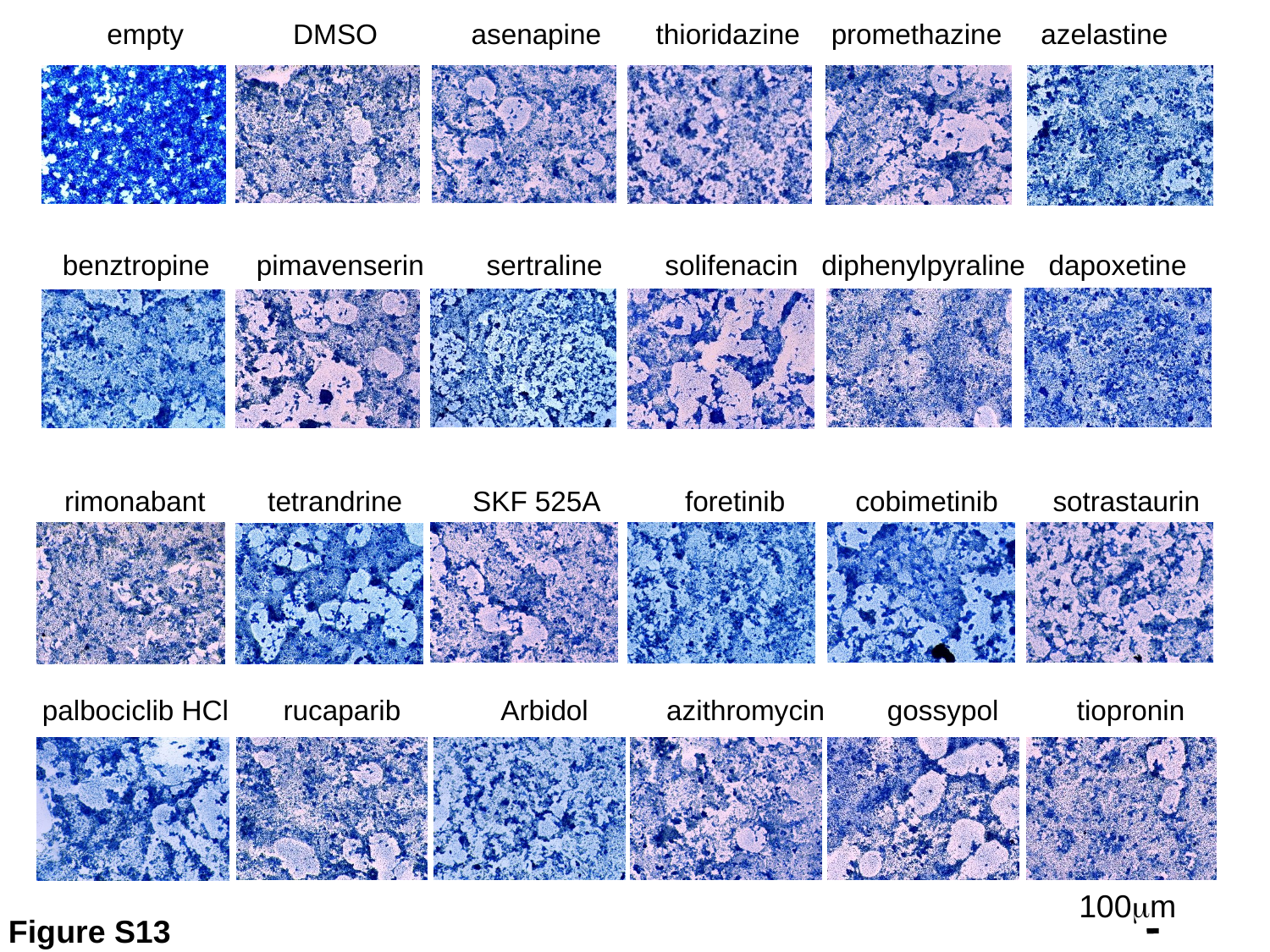

empty DMSO asenapine thioridazine promethazine azelastine
benztropine pimavenserin sertraline solifenacin diphenylpyraline dapoxetine
rimonabant tetrandrine SKF 525A foretinib cobimetinib sotrastaurin
palbociclib HCl rucaparib Arbidol azithromycin gossypol tiopronin
100mm
Figure S13
