# Supplementary material for: Fusion assays for screening of fusion inhibitors targeting SARS-CoV-2 entry and syncytia formation
Source: Front Pharmacol. 2022 Nov 11;13:1007527. doi: 10.3389/fphar.2022.1007527 (PMC9691968; doi:10.3389/fphar.2022.1007527)
Supplement: Supplementary file 13 [file DataSheet1.docx]

**Figure S1 Standard curve of luciferase activity.** Ten-fold serial dilutions of recombinant luciferase were used to construct a standard curve of the luciferase assay. **(A)** A representation of absolute luciferase activity. **(B)** Relative luciferase activity of mean+/-SD of three repeats.

**Figure S2 Expression of ACE2 and spike protein**. **(A)** Western blot of ACE2 from 2.5x10^5^ 293T, 293T-ACE2 and 293T-ACE2-TMPRSS2 cells together with serial dilutions of recombinant ACE2 protein. **(B)** Western blot of SARS-CoV-2 spike protein from 2.5x10^5^ empty vector- and spike protein-transfected 293T cells together with serial dilutions of recombinant spike protein. A longer exposure of the empty vector- and spike protein-transfected 293T cells is presented on the right-hand panel. S0=full-length uncleaved spike protein; S1 & S2 are the two sub-units of the spike protein. TGX stain-free gels show equal loading of total proteins.

**Figure S3 Optimal cell seeding densities for luciferase assays.** Donor 293T cells were transfected with empty vector (negative control) or the severe acute respiratory syndrome coronavirus 2 spike protein (SARS-2-S) or the vesicular stomatitis virus G glycoprotein (VSV-G), respectively, together with a plasmid encoding the T7 polymerase. Target 293T cells were transfected with the luciferase and β-galactosidase reporter genes. Donor and target cells were co-cultured at different cell densities for 16h. VSV-G co-cultures were treated with pH5 for 2min and incubated for a further 5h. Fusion activity was measured as luciferase activity normalized against β-galactosidase activity and expressed as a ratio to their respective vector control under the respective fusion condition.

**Figure S4 Morphological fusion assay.** 50,000 293T-ACE2-TMPRSS2 pre-incubated with 10μM of individual drugs/solvents were co-cultured with 50,000 donor 293T cells transfected with an empty vector or the SARS-CoV-2 spike protein (SARS-2-S). Cells were fixed and stained with methylene blue. Bright-field images are of the same magnification x40 and scale.

**Figure S5 Optimal treatment time for fusion triggers.** Donor 293T cells were transfected with empty vector (negative control) or the severe acute respiratory syndrome coronavirus 2 spike protein (SARS-2-S), respectively, together with a plasmid encoding the T7 polymerase. Target 293T cells were transfected with the luciferase and β-galactosidase reporter genes. 19,000 each of donor and target cells were co-cultured for 16h, treated as indicated for 2, 5, 10 and 15mi and incubated for a further 5h. Fusion activity was measured as luciferase activity normalized against β-galactosidase activity and expressed as a ratio to their respective vector control at the respective fusion condition.

**Figure S6 Physiological pH-triggered fusion time-course.** 19,000 target cells (293T-ACE2, 293T-ACE2-TMPRSS2) transfected with the luciferase and β-galactosidase reporter genes were co-cultured with 19,000 donor 293T cells transfected with an empty vector or the severe acute respiratory syndrome coronavirus 2 (SARS-CoV-2) spike protein together with a plasmid encoding the T7 polymerase at pH7.4 over a time-course of 24h. **(A)** Photomicrographs illustrate the kinetics of morphological fusion. Due to space limitation only the photomicrographs of co-culture of empty vector-transfected cells at 24h are shown. Bright-field images are of the same magnification x100 and scale. **(B) (left)** β-galactosidase and luciferase activities in SARS-2-S fusion cells were expressed as a ratio to their respective empty vector control at the same time-point. **(right)** Fusion activity was measured as luciferase activity normalized against β-galactosidase activity and expressed as a ratio to their respective empty vector control at the same time-point.

**Figure S7 Time-course of fusion triggered by pH7+trypsin, pH5 and pH5+trypsin.** 19,000 target cells (293T-ACE2, 293T-ACE2-TMPRSS2) transfected with the luciferase and β-galactosidase reporter genes were co-cultured with 19,000 donor 293T cells transfected with an empty vector or the severe acute respiratory syndrome coronavirus 2 (SARS-CoV-2) spike protein together with a plasmid encoding the T7 polymerase in 10% fetal calf serum (FCS)/DMEM for 16h. The co-cultures were treated with pH7+trypsin, pH5 or pH5+trypsin for 2min and then incubated in 10% FCS/DMEM over a time-course of 7h. **(A)** Photomicrographs illustrate the kinetics of morphological fusion. Due to space limitation only the photomicrographs of co-culture of empty vector-transfected cells at 7h are shown. Bright-field images are of the same magnification x100 and scale. **(B) (left)** β-galactosidase and luciferase activities in SARS-2-S fusion cells were expressed as a ratio to their respective empty vector control at the same time-point. **(right)** Fusion activity was measured as luciferase activity normalized against β-galactosidase activity and expressed as a ratio to their respective empty vector control at the same time-point.

**Figure S8 Morphology of cell fusion at pH7.4.** 19,000 target cells (293T-ACE2, 293T-ACE2-TMPRSS2) transfected with the luciferase and β-galactosidase reporter genes were co-cultured with 19,000 donor 293T cells transfected with an empty vector or the severe acute respiratory syndrome coronavirus 2 (SARS-CoV-2) spike protein together with a plasmid encoding the T7 polymerase. The co-cultures were treated at pH7.4. Photomicrographs illustrate intact single cells and syncytia (arrowheads). Bright-field images are of the same magnification x100 and scale and are representative of 2 repeats.

**Figure S9 Morphology of cell fusion at pH5.** 19,000 target cells (293T-ACE2, 293T-ACE2-TMPRSS2) transfected with the luciferase and β-galactosidase reporter genes were co-cultured with 19,000 donor 293T cells transfected with an empty vector or the severe acute respiratory syndrome coronavirus 2 (SARS-CoV-2) spike protein together with a plasmid encoding the T7 polymerase. The co-cultures were treated at pH5. Photomicrographs illustrate intact single cells and syncytia (arrowheads). Bright-field images are of the same magnification x100 and scale and are representative of 2 repeats.

**Figure S10 Morphology of cell fusion at pH7.4+trypsin.** 19,000 target cells (293T-ACE2, 293T-ACE2-TMPRSS2) transfected with the luciferase and β-galactosidase reporter genes were co-cultured with 19,000 donor 293T cells transfected with an empty vector or the severe acute respiratory syndrome coronavirus 2 (SARS-CoV-2) spike protein together with a plasmid encoding the T7 polymerase. The co-cultures were treated at pH7.4+trypsin. Photomicrographs illustrate intact single cells and syncytia (arrowheads). Bright-field images are of the same magnification x100 and scale and are representative of 2 repeats.

**Figure S11 Morphology of cell fusion at pH5+trypsin.** 19,000 target cells (293T-ACE2, 293T-ACE2-TMPRSS2) transfected with the luciferase and β-galactosidase reporter genes were co-cultured with 19,000 donor 293T cells transfected with an empty vector or the severe acute respiratory syndrome coronavirus 2 (SARS-CoV-2) spike protein together with a plasmid encoding the T7 polymerase. The co-cultures were treated at pH5+trypsin. Photomicrographs illustrate intact single cells and syncytia (arrowheads). Bright-field images are of the same magnification x100 and scale and are representative of 2 repeats.

**Figure S12 Statistical depiction of Figure 6: Drug inhibition profiles of different modes of fusion.** 19,000 target cells (293T-ACE2, 293T-ACE2-TMPRSS2) transfected with the luciferase and β-galactosidase reporter genes were pre-incubated with 10μM of individual drugs before co-cultured with 19,000 donor 293T cells transfected with the severe acute respiratory syndrome coronavirus 2 (SARS-CoV-2) spike protein together with a plasmid encoding the T7 polymerase. The co-cultures were treated as indicated. Fusion activity was measured as luciferase activity normalized against β-galactosidase activity and expressed as a ratio to the solvent control in the respective cell type and fusion condition which is set as 1. Data are presented as mean +/- SD of 3 repeats. *p<0.05, **p<0.01 and ***p<0.001. Pharmacological classes of drugs are colour-coded.

**Fig.S13 Fusion cell morphology.** 50,000 293T-ACE2-TMPRSS2 pre-incubated with 10μM of individual drugs were co-cultured with 50,000 donor 293T cells transfected with an empty vector or the SARS-CoV-2 spike protein. The co-cultures were treated as indicated. Cells were fixed and stained with methylene blue. Bright-field images are of the same magnification x100 and scale.
